# A comparison of methods for the optimal recovery of the human fecal virome

**DOI:** 10.1093/ismeco/ycag090

**Published:** 2026-04-11

**Authors:** Loretta De Chiara, Ryan Doughty, Nuria Estévez-Gómez, Pilar Gallego-García, Pilar Alvariño, Astrid Díez-Martín, Pedro Dávila Piñón, Todd J Treangen, Joaquín Cubiella, David Posada

**Affiliations:** CINBIO, Universidade de Vigo, Vigo, 36310, Spain; Galicia Sur Health Research Institute (IIS Galicia Sur), SERGAS-UVIGO, Vigo, 36312, Spain; Department of Computer Science, Rice University, Houston, TX 77005-1892, United States; CINBIO, Universidade de Vigo, Vigo, 36310, Spain; Galicia Sur Health Research Institute (IIS Galicia Sur), SERGAS-UVIGO, Vigo, 36312, Spain; CINBIO, Universidade de Vigo, Vigo, 36310, Spain; Galicia Sur Health Research Institute (IIS Galicia Sur), SERGAS-UVIGO, Vigo, 36312, Spain; CINBIO, Universidade de Vigo, Vigo, 36310, Spain; Galicia Sur Health Research Institute (IIS Galicia Sur), SERGAS-UVIGO, Vigo, 36312, Spain; Research Group in Gastrointestinal Oncology-Ourense, CIBERehd, Ourense, 32005, Spain; Research Group in Gastrointestinal Oncology-Ourense, CIBERehd, Ourense, 32005, Spain; Department of Computer Science, Rice University, Houston, TX 77005-1892, United States; Department of Bioengineering, Rice University, Houston, TX 77005-1892, United States; Ken Kennedy Institute, Rice University, Houston, TX 77005-1892, United States; Department of Gastroenterology, Health Area of Ourense, Verín and Barco de Valdeorras, Ourense, 32005, Spain; CINBIO, Universidade de Vigo, Vigo, 36310, Spain; Galicia Sur Health Research Institute (IIS Galicia Sur), SERGAS-UVIGO, Vigo, 36312, Spain

**Keywords:** virome, metagenomics, protocol, human feces

## Abstract

Human virome research is gaining increasing attention as viruses are recognized as critical modulators of microbial communities and human health. Viral metagenomics, however, faces unique challenges, including the low abundance and diversity of viruses in biological samples, the absence of universal marker genes, and biases introduced by experimental protocols. While various virome protocols have been benchmarked using viral particles or nucleic acids from mock communities, these approaches often fail to capture the complexity and heterogeneity of natural viromes. In this study, we systematically evaluated modifications to key methodological steps in the metagenomic analysis of human fecal samples, including viral enrichment, nucleic acid extraction, genome amplification, and library preparation. Using gold-standard bioinformatic approaches on sequencing datasets generated after amplification, we assessed the impact of these modifications on relative viral taxonomic assignment, contig quality, richness, diversity, and inferred genome structure. Our findings reveal striking trade-offs between recovery of viral genomes and retention of nonviral sequences, demonstrating how methodological choices can shape the inferred virome composition. Based on these observations, we propose an optimized protocol that enhances viral genome recovery while reducing contamination from nonviral sequences. This refined workflow provides a more robust and reliable framework for gut virome studies, paving the way for a deeper exploration of the role of viruses in human health and microbial ecosystems.

## Introduction

The human gut virome is now recognized as a critical component of the intestinal microbiome, primarily dominated by phages that infect and shape bacterial communities [1, 2]. Despite its central role, the gut virome is much less well characterized than the bacteriome. Viruses are difficult to study due to their variability in genome type, strandedness, structure and size, replication strategies, host range, and morphology. Shotgun metagenomics has revolutionized virology by significantly expanding our knowledge of viral diversity [3], but important limitations remain: many viruses occur at low abundance, display high sequence divergence, and lack universal marker genes [4]. Taxonomic classification is particularly challenging largely because methods rely on sequence alignment and reference databases in which viral representation is still sparse [5, 6]. Early virome studies left more than 90% of viral reads unclassified, a problem often called “viral dark matter” [7–9]. Recent updates by the International Committee on Taxonomy of Viruses (ICTV) and advances in virome bioinformatics have begun to reduce this dark matter, enabling more robust assignments at higher taxonomic ranks for an increasing fraction of viral sequences [10].

Protocols for viral metagenomics date back more than a decade, initially using 454 pyrosequencing, then later Illumina short-read platforms, and more recently long-read technologies [11–13]. Shotgun virome metagenomics includes several critical steps: purification of viral particles to reduce host and microbial contaminants, extraction of viral nucleic acids, and preparation of sequencing libraries, with genome amplification often performed when viral nucleic acid yield is low. When sufficient viral DNA is available, unamplified viromes can be generated from human stool samples [14–16]. Each step is designed to maximize viral genome recovery while minimizing background noise [1, 17]. Most studies rely on untargeted, shotgun approaches to detect both known and novel viruses; however, capture-based or probe-enrichment strategies have also been adopted to increase sensitivity for known viral targets or low-abundance taxa [18, 19].

Laboratory methods can introduce systematic biases in genome representation, potentially affecting virome characterization [20]. Benchmarking and comparative studies have been instrumental in highlighting how extraction and amplification methods influence viral recovery and diversity estimates [21–27]. However, most of these evaluations use highly simplified systems—mock communities, spiked samples containing only a few DNA virus species at high concentrations, or pooled viral nucleic acids, which do not reflect the complexity, low viral abundance, and host/microbial background of real biological specimens. RNA viruses are also frequently underrepresented or omitted entirely, either by design or because protocols fail to recover them effectively, further limiting our ability to capture the full complexity of natural viromes. While benchmarking studies on environmental and clinical samples yielded valuable insights, their findings cannot be directly extrapolated to human feces. Human stool presents a distinct set of challenges: it typically yields low amounts of viral nucleic acids, contains abundant bacterial DNA, and harbors various enzymatic inhibitors and complex organic matter that interfere with particle purification, nucleic acid extraction, amplification, and library preparation.

As a result, protocols optimized for seawater, soil, wastewater, or even other clinical specimens (e.g. saliva, blood, respiratory samples) [24, 28–31] often perform poorly on stool. Moreover, most prior evaluations have examined isolated workflow steps (viral enrichment, nucleic acid extraction, or amplification) and often used quantitative real-time PCR (qPCR) as a proxy for performance, which does not fully reflect the complexity of viral metagenomic data. Comprehensive, feces-specific benchmarking using metagenomic read- and assembly-based metrics is therefore essential to identify workflows that genuinely improve virome recovery and characterization.

Our study builds upon previous benchmarking efforts by delivering an integrated, stepwise evaluation of all major workflow stages for virome analysis using human fecal samples instead of mock or environmental communities. We systematically compared multiple shotgun metagenomics protocols across four critical steps: (i) viral enrichment, (ii) DNA and RNA extraction, (iii) genome amplification, and (iv) sequencing library preparation. Because every protocol required an amplification step, the resulting datasets are not quantitative; therefore, comparisons emphasize detection and characterization of viral taxa rather than absolute abundance. This design enabled us to thoroughly quantify how methodological choices influence taxonomic read assignments, contig quality, viral richness and diversity, and genome structure. From these results, we derive an optimized protocol that improves viral genome recovery while minimizing nonviral contamination, providing a practical framework for more accurate, reproducible gut virome studies.

## Materials and methods

### Study design

**Figure 1:**
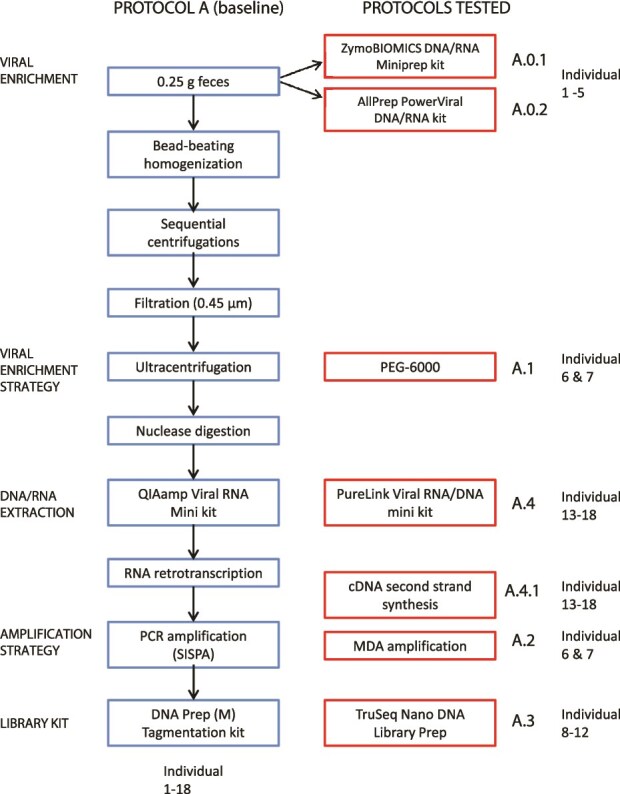
Workflow for shotgun metagenomics sequencing of the human fecal virome. The baseline protocol (Protocol A) is shown in blue on the left, while the modifications tested are highlighted in red on the right. Each modification was assessed separately, keeping all other steps consistent with Protocol A. Modifications tested: Protocol A.0.1 and A.0.2: direct nucleic acid extraction without viral enrichment, followed by RNA retrotranscription; Protocol A.1: viral precipitation with PEG-6000 (instead of ultracentrifugation); Protocol A.2: MDA amplification (instead of PCR-SISPA with pseudo-degenerate oligonucleotides); Protocol A.3: library preparation using TruSeq Nano DNA Library Prep (instead of DNA Prep (M) Tagmentation kit); Protocol A.4: DNA/RNA extraction using PureLink Viral RNA/DNA Mini kit (instead of QIAmp Viral RNA Mini kit); Protocol A.4.1: same as Protocol A.4, with the addition of second-strand cDNA synthesis (instead of QIAmp Viral RNA Mini kit without second-strand cDNA synthesis). The individual used for each protocol is shown.

We established a baseline protocol (Protocol A) as the starting point for optimization. This protocol combines sequential low-speed centrifugations to remove debris and cells, filtration to exclude larger particles, ultracentrifugation to concentrate viral particles, and nuclease treatment (DNase and RNase) to degrade unencapsidated nucleic acids. Following enrichment, Protocol A includes RNA and DNA extraction, reverse transcription (RT) of RNA into cDNA, amplification using PCR with pseudo-degenerate oligonucleotides (single primer amplification, SISPA), and sequencing library construction using a tagmentation-based approach (Fig. 1).

From this baseline, we evaluated six methodological modifications to assess their impact on virome recovery and composition:

1. Direct nucleic acid extraction without prior enrichment (Protocols A.0.1 and A.0.2).
2. Polyethylene glycol (PEG) precipitation to concentrate viral particles instead of ultracentrifugation (Protocol A.1).
3. Multiple displacement amplification (MDA) as an alternative to PCR-SISPA for genome amplification (Protocol A.2).
4. Ligation-based library preparation instead of tagmentation (Protocol A.3).
5. An alternative nucleic acid extraction kit to test extraction chemistry (Protocol A.4).
6. Optional second-strand synthesis of cDNA to evaluate the effect of converting single-stranded cDNA to double-stranded form before amplification (Protocol A.4.1).

### Samples

We collected stool samples from 18 adult volunteers who had not taken antibiotics for at least 2 months. Participants were recruited at the Complexo Hospitalario Universitario de Ourense, and specimens were provided by the Biobank of the Galicia Sur Health Research Institute (IISGS). All participants gave written informed consent; sample handling preserved anonymity and complied with Spanish regulations and the Declaration of Helsinki. The study protocol was approved by the Ethics Committee for Clinical Research of Galicia (2021/134).

Each stool sample was processed with the baseline Protocol A, and subsets of samples were allocated to test alternative methods as follows. Five individuals (IDs 1–5; 3 men, 2 women) were used to compare Protocol A with the two direct-extraction variants (A.0.1 and A.0.2). Two individuals (IDs 6–7; 2 men) were used to compare Protocol A with PEG precipitation (A.1) and MDA (A.2). Five individuals (IDs 8–12; 2 men, 3 women) were used to compare Protocol A with ligation-based library preparation (A.3). Six individuals (IDs 13–18; 4 men, 2 women) were used to compare Protocol A with an alternative extraction kit (A.4) and the optional cDNA second-strand synthesis (A.4.1). All 18 samples were therefore processed with Protocol A, enabling within-sample comparisons across the tested modifications.

### Homogenization

We thawed and homogenized the fecal samples to a uniform consistency. For each protocol, we transferred 0.25 g of stool into ZR BashingBeads Lysis Tubes (0.1/0.5 mm beads; Zymo Research, CA, USA) and added 750 μl of DNA/RNA Shield (Zymo Research, CA, USA). We then bead-beated the samples for 10 min on a Vortex-Genie 2 (Scientific Industries, NY, USA) to disrupt the cells and release the viral particles.

### Enrichment and precipitation of viral particles

We centrifuged the homogenized fecal samples (14 000 g, 30 s, 8°C) and recovered 400 μl of supernatant, to which we added 800 μl of Hanks′ Balanced Salt Solution (HBSS; Sigma, MA, USA). To remove large particulate matter, we performed three sequential centrifugations (10 000 g, 2 min, 8°C) to sediment large fecal aggregates. One mL of the clarified supernatant was passed through a 0.45 μm syringe filter and then concentrated either by ultracentrifugation (Protocol A) or by PEG-based precipitation (Protocol A.1).

For ultracentrifugation, we transferred the filtered suspension into 6 ml Quick-Seal® Bell-Top Polypropylene Tubes (Beckman Coulter Life Sciences, CA, USA), topped them with HBSS, heat-sealed the tubes, and centrifuged at 750 000 g for 1 h at 8°C in an Optima™ XPN-100 ultracentrifuge with a 100 Ti fixed-angle rotor (Beckman Coulter Life Sciences, CA, USA). In all cases, we observed a visible pellet following ultracentrifugation. Although pellets were visible for our samples, we marked the outermost edge of each tube in the rotor orientation to guide careful removal of supernatant and minimize pellet loss when the pellet was small. We resuspended each pellet in 500 μl of HBSS, and stored the suspension at −80°C.

For PEG-based precipitation, we adjusted the filtered supernatant to ~1 M NaCl by adding 5 M NaCl (Promega, Madison, WI, USA) and then added PEG-6000 (Calbiochem, CA, USA) to 10% (w/v). After gentle inversion to mix, we incubated the tubes overnight at 4°C, pelleted the PEG-virus complexes (1200 g, 20 min, 4°C), resuspended the pellet in 500 μl HBSS, and stored at −80°C. Although the PEG pellet was visible in all cases, viscous residual material in the supernatant made pelleting and complete recovery challenging, which reduced reproducibility. To remove unencapsidated nucleic acids, we treated 120 μl of the viral suspension with 4.8 U Turbo DNase, 1X Turbo DNase buffer, 4 U RNase A, 160 U RNase T1 (Invitrogen, MA, USA), and 25 U benzonase (>90% purity; Millipore, MA, USA). We incubated the mixture 1.5 h at 37°C before proceeding to nucleic acid extraction.

### Nucleic acid extraction

We extracted viral DNA and RNA from the digested product using commercial spin-column kits according to the manufacturers’ protocols, without adding carrier RNA. For the main workflow (Protocol A), we used the QIAamp Viral RNA Mini kit (Qiagen, Hilden, Germany). For Protocols A.4 and A.4.1, we used the PureLink Viral RNA/DNA Mini kit (Invitrogen, Waltham, MA, USA). For Protocols A.0.1 and A.0.2, we evaluated two additional kits: ZymoBIOMICS DNA/RNA Miniprep kit (Zymo Research, Irvine, CA, USA) and AllPrep PowerViral DNA/RNA kit (Qiagen, Hilden, Germany). For both kits, we processed 0.25 g of feces and used the supplied beads for bead-beating. For the ZymoBIOMICS kit, we followed the total nucleic acid purification protocol. For the AllPrep PowerViral kit, we added β-mercaptoethanol to improve viral RNA recovery. In all cases, we eluted the nucleic acids in 50 μl of nuclease-free water (NFW) and stored them at −80°C.

### Retrotranscription of RNA viruses and cDNA second-strand synthesis

We used 11 μl of nucleic acid extract as template for RT with SuperScript IV (Invitrogen, MA, USA). Each RT reaction contained 1 μl of 50 μM pseudo-degenerate primer D2_8N (5’-AAGCTAAGACGGCGGTTCGGNNNNNNNN-3′) [32], which provides eight random nucleotides for random priming attached to a 20-nucleotide defined tag. The final reaction volume was 20 μl.

For Protocol A.4.1, we converted the single-stranded cDNA to double-stranded cDNA using the Second Strand cDNA Synthesis kit (Invitrogen, MA, USA). We then treated the resulting double-stranded cDNA with RNase I to remove any residual RNA and purified the product using the QIAquick PCR Purification Kit (Qiagen, Hilden, Germany).

### Genomic amplification

We evaluated two genomic amplification strategies, PCR-SISPA (Protocol A) and MDA (Protocol A.2). For PCR-SISPA, we used 8 μl of the RT product (or double-stranded cDNA for Protocol A.4.1) as template in a 50 μl reaction with Q5 High-Fidelity DNA Polymerase (New England Biolabs, MA, USA) and 3 μl of 20 μM D2 primer (5’-AAGCTAAGACGGCGGTTCGG-3′) [32]. Thermal cycling was performed as follows: initial denaturation at 98°C for 1 min; 30 cycles of 98°C for 10 s, 55°C for 30 s, and 72°C for 45 s; final extension at 72°C for 2 min, and cooling to 4°C.

For MDA, we amplified 1 μl of the reverse-transcribed product using the illustra GenomiPhi v2 DNA Amplification kit (GE Healthcare, IL, USA). To minimize amplification bias inherent to MDA, we performed three independent reactions per sample and pooled the products prior to downstream processing.

We quantified the amplified products with the Qubit 3.0 dsDNA BR Assay kit (Thermo Fisher Scientific, MA, USA) and evaluated fragment size distribution with the 2200 TapeStation D1000 kit (Agilent Technologies, CA, USA).

### Library preparation and sequencing

We prepared sequencing libraries from the PCR-SISPA products using two Illumina workflows, a tagmentation-based approach (DNA Prep (M) Tagmentation kit; formerly Nextera DNA Flex; Protocol A) and a ligation-based approach (TruSeq Nano DNA Library Prep; Protocol A.3).

For the DNA Prep (M) tagmentation libraries (Protocol A), we used ~125 ng input DNA and performed reactions at 1/4 of the manufacturer’s recommended volumes to conserve reagents. Libraries were indexed with Nextera DNA CD Indexes (Illumina, CA, USA) and amplified for five PCR cycles as recommended for reduced input. For TruSeq Nano libraries (Protocol A.3), we used 100 ng input DNA, omitting DNA fragmentation, end-repair, and size-selection steps to preserve fragment integrity; we proceeded directly to 3′ adenylation and adapter ligation, and indexed libraries with TruSeq DNA CD Indexes (Illumina, CA, USA).

We quantified all libraries as described in the previous section and sequenced them on an Illumina MiniSeq using the MiniSeq High Output Reagent kit (PE150 reads for DNA Prep and SE150 reads for TruSeq). We performed five sequencing runs in a multiplexing configuration as follows: 17-plex (Protocols A, A.0.1, and A.0.2); 90-plex (A, A.1, and A.2); 6-plex x 2 (A and A.3); and 25-plex (A, A.4, and A.4.1). Pooling was adjusted to target balanced read depth across samples.

### Bioinformatics quality control and preprocessing

We implemented a standardized preprocessing pipeline to clean and assess raw reads prior to virome analyses. We trimmed adapters, low-quality bases, and short reads with fastp [33] (parameters: -l 50 -y 3 -W 4 -M 20 -x). We removed human reads by aligning filtered reads to the GRCh38 (hg38) reference genome with Bowtie2 v2.5.4 [34]. We monitored read quality at each step with FastQC [35] and aggregated reports with MultiQC [36] to track per-base quality, duplication, GC content, adapter content, and read length distributions. We used ViromeQC v1.0 [37] to estimate viral enrichment scores and quantify bacterial contamination.

### Assembly and identification of viral contigs

We assembled metagenomic contigs for each sample with MEGAHIT v1.2.9 [38] using default parameter values and identified viral contigs from these assemblies using geNomad v1.9.3 [39] using default parameters and applying a minimum contig length filter of 1000 bp. We assessed viral contig quality and completeness with CheckV v1.0.3 [40] and retained only contigs classified as medium-, high-quality, or complete genomes for downstream viral operational taxonomic unit (vOTU) analyses.

### Viral operational taxonomic unit clustering, taxonomic and structural annotations, read mapping, and compositional calculations

Because we recovered relatively few high-quality viral contigs in our study, we wanted to ensure that we were able to recover a broader diversity of low-abundance phage genomes that were unable to be assembled directly. Therefore, we combined high-quality and complete genomes assembled from our datasets with high-quality genomes from the Unified Human Gut Virome (UHGV, [41], accessed 18 December 2025) and reclustered the combined set into a final set of vOTUs. Clustering was performed using all-vs-all blastn [42] and custom scripts from CheckV [40] (scripts/ani_calc.py, scripts/ani_clust.py) requiring 95% average nucleotide identity (ANI) over at least 85% of the length of the shortest sequence. This clustering threshold is widely used to approximate species-level viral taxonomy, and vOTUs were therefore treated as species-level operational units throughout the study. For each cluster, we retained the longest genome as the representative sequence.

For abundance estimation, we mapped quality-filtered reads to the complete representative vOTU set using Bowtie2 [34], retaining only concordantly mapped paired reads (when paired-end data were available) and primary alignments. We generated per-vOTU read counts with samtools idxstats [43] and further normalized counts by sequencing library size to get abundance measures for comparative analyses.

To obtain comparable ICTV annotations for the genomes assembled in the study, we annotated each study-contig with the UHGV classifier to infer putative ICTV lineages, predicted host lineages, and lifestyle (temperate vs lytic). We then mapped those ICTV lineages to current reference metadata (ICTV Virus Metadata Resource (VMR_MSL39_v1 [44]; VMR_MSL40.v2, release date 13 October 2025) and ViralZone [45]) to extract genome composition and structural traits (e.g. envelope presence, particle size) (accessed December 2025). Genome types were grouped into higher-order categories (ssDNA, ssRNA, positive and negative sense, dsDNA, dsRNA, and reverse-transcribing viruses) for summary analyses. We used this information to obtain higher-level compositional profiles by aggregating vOTU abundances by ICTV ranks (family, class, order, etc.) and by genome composition or structural trait. For each protocol, we then calculated the mean percentages for each viral genome type and of the structural characteristic to summarize central tendencies while minimizing the influence of outliers.

### Richness and diversity metrics

We assessed viral diversity by applying read-count and abundance thresholds to limit spurious low-support taxa and to evaluate the contribution of rare vs abundant vOTUs. For read thresholds *n* ranging from 0 to 300 000 in increments of 1000, we removed vOTUs with fewer than *n* supporting reads and computed species-level richness as well as Shannon [46] and Simpson [47] diversity indices on the remaining set. We repeated this process using abundances, filtering from 0% to 100% in increments of 1%. This threshold sweep produces a diversity curve analogous to a cumulative abundance filter and allows us to visualize how diversity estimates change as lower-abundance signals are progressively excluded. We interpreted alpha diversity metrics strictly in a relative framework because all comparisons use the same biological samples processed with different protocols. In the absence of an absolute reference for viral species richness in human fecal samples, these metrics therefore indicate the relative breadth and robustness of viral recovery achieved by each protocol. We also repeated this experiment over thresholds *n* ranging from 0 to 50 000 reads, with increments of 500 to get more resolution on lower-abundance taxa, as well as from 0% to 10% with increments of 0.1%.

### Statistical analyses

We performed statistical analyses using nonparametric and compositionally aware methods appropriate for our data distributions and experimental design. To compare read assignments, assembly metrics, and other continuous sample-level outcomes between each experimental protocol and Protocol A, we used the Mann–Whitney *U*-test. For viral diversity across read thresholds, we followed the approach described in Hristova and Wimley [48], summarizing diversity-threshold curves and testing overall differences between protocols with permutation-based curve comparisons (*n* = 10 000 permutations). To compare viral composition between protocols, we converted family-level viral read counts to relative abundances and applied a centered log-ratio transformation to account for compositionality [49].

## Results

We established a baseline fecal virome workflow (Protocol A; Fig. 1) as the reference and systematically evaluated targeted modifications at key methodological steps. We compared the relative performance of the different protocols across multiple metrics, including taxonomic read assignments, assembly outcomes and contig quality, viral diversity, and the distribution of genome types and structural traits assigned to the viral families identified.

### Overview of protocol outcomes

We compared read yields and taxonomic assignments across protocols; detailed counts before and after QC and mappings to human, viral, and unassigned categories are provided in Tables S1 and S2. After filtering and quality control, 108 viral contigs passed our thresholds and were carried forward for further characterization and clustering. The final set of representative sequences comprised 57 contigs originating from this study plus 57 357 from the UHGV.

**Figure 2:**
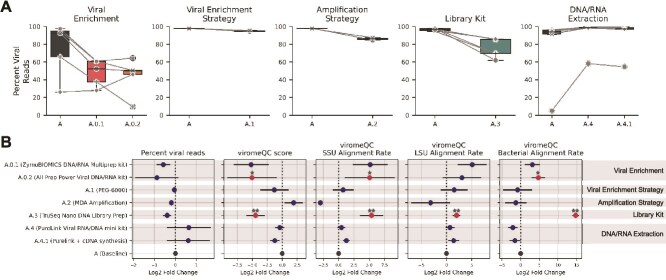
Overview of the outcomes of the tested protocols. (A) Boxplots showing the percentage of viral reads mapping to vOTUs. The central line represents the median, box limits indicate Q1 and Q3, and whiskers extend to the lowest and highest nonoutlier values (Q1–1.5 IQR and Q3 + 1.5 IQR). Individual sample data points are also displayed. (B) Mean and 95% confidence interval (CI) of the log2 fold-change in viral reads mapping to vOTUs and the ViromeQC score, together with the alignment rates to the small subunit ribosomal RNA genes (SSU), large subunit ribosomal RNA genes (LSU), and bacterial markers (31 bacterial genes). Each modified protocol was compared to Protocol A within its respective set of processed samples. Significant differences (*P* < =.05, |fold change| > 1) are labelled in red and ^*^  *P* < .05; ^**^  *P* < .01.

Omitting viral enrichment (A.0.1 and A.0.2) substantially reduced viral recovery, producing a mean decrease of 28% in the proportion of reads mapping to vOTUs relative to the enriched baseline (Protocol A) and was associated with lower ViromeQC scores and higher SSU, LSU, and bacterial alignment rates (Mann–Whitney *U*-test *P* < .05 for A.0.2) (Fig. 2B).

Between enrichment approaches, ultracentrifugation (Protocol A) slightly outperformed PEG-based purification (A.1) across vOTU mapping percentage, ViromeQC score, and SSU and LSU alignment rates (Fig. 2). The amplification strategy had a subtle impact on the viral read fraction: PCR-SISPA (A) yielded 98.06% viral reads vs 86.9% for MDA (A.2). Despite a lower viral recovery, MDA produced the highest ViromeQC scores and lower alignment rates to SSU, LSU, and bacterial markers (SSU: 0.0006 vs. 0.0047; LSU: 0.0048 vs. 0.0260; bacterial markers: 0.0004 vs. 0.0021 for MDA vs. PCR-SISPA).

Library construction also influenced recovery: ligation-based TruSeq libraries (A.3) showed a 0.77-fold reduction in reads mapping to vOTUs compared with tagmentation DNA Prep libraries (A) (Mann–Whitney *U* test, *P* < .05), consistent with lower ViromeQC scores and higher SSU, LSU, and bacterial alignments (Fig. 2B).

Finally, extraction chemistry had a marked impact: PureLink kit (A.4) outperformed the widely used QIAamp kit (A), yielding a mean 2.69-fold higher proportion of reads mapping to vOTUs (Fig. 2A), and a mean 2.58-fold increase after adding second-strand cDNA synthesis (A.4.1). However, this improvement was not consistently reflected in the ViromeQC metrics (Fig. 2B).

### Viral assembly quality

**Figure 3:**
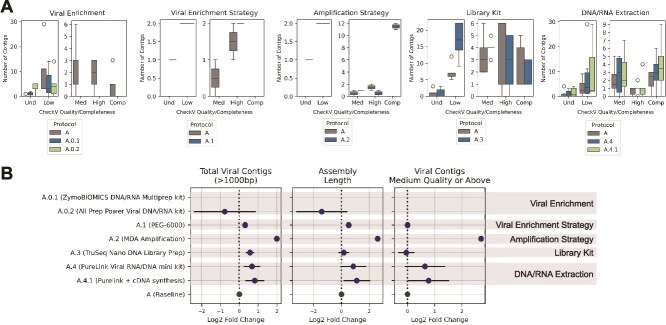
Quality of viral assemblies across the protocols tested. (A) Boxplots showing the number of contigs >1000 bp assembled using MEGAHIT, identified as viral by geNomad and categorized by CheckV quality and completeness scores: Und: undetermined quality (no completeness estimate available), low quality: <50% completeness, medium (Med) quality: 50-90% completeness, high quality: ≥90% completeness, and complete genomes (Comp). The central line represents the median, box limits indicate the Q1 and Q3 quartiles, and whiskers extend to Q1–1.5 IQR and Q3 + 1.5 IQR, marking the lowest and highest nonoutlier values. (B) Mean and 95% CI of the log2 fold-change in the number of contigs >1000 bp, assembly length (total nucleotides in those contigs), and total number of contigs classified as medium, high-quality, or complete. Each modified protocol was compared to Protocol A within its respective set of processed samples. Protocols A.0.1 and A.0.2 do not have contigs at medium or greater quality.

Viral enrichment had a major impact on assembly: samples processed without enrichment (A.0.1 and A.0.2) produced less and shorter viral contigs than the enriched baseline (A) (Fig. 3A and B). All viral contigs from the nonenriched protocols were classified by CheckV as undetermined or low-quality. In contrast, Protocol A yielded medium- and high-quality contigs, and several complete viral genomes.

Comparing enrichment methods, PEG precipitation (A.1) generated more and longer viral contigs than ultracentrifugation (A) (median contig length 2724 bp vs 1450 bp), with a higher fraction of undetermined/low-quality contigs but a comparable number of high-quality contigs. The amplification strategy also influenced the assemblies. MDA (A.2) produced more contigs, a greater proportion >1 Kb and more complete genomes than PCR-SISPA (A) (Fig. 3). Notably, CheckV annotated direct terminal repeats (DTRs) in all complete genomes recovered from MDA samples, consistent with circular or long terminal repeat-bounded genome structures [40].

Library preparation also affected assembly. Ligation-based libraries (A.3) returned more viral contigs than tagmentation libraries (A) (Mann–Whitney *U*-test *P* < .05) but similar assembly length. However, tagmentation libraries produced a higher number of contigs classified as medium, high-quality, or complete genomes.

Finally, nucleic acid extraction chemistry was relevant. PureLink (A.4 and A.4.1) produced more and longer viral contigs and a higher proportion of medium and high-quality contigs and complete genomes than QIAamp (A).

### Richness and alpha diversity metrics

**Figure 4:**
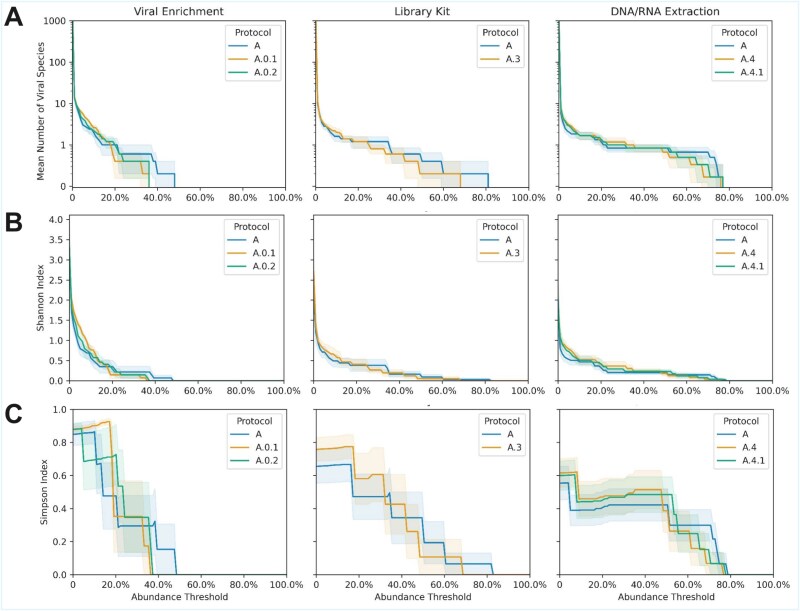
Richness and diversity metrics of viral taxonomic assignments across protocols. Metrics were calculated using vOTUs, treated as species-level units, and calculated at increasing abundance thresholds, disregarding any taxa below the required abundance. Cutoffs from 0%–100% with step sizes of 1% were calculated. (A) Species-level richness, defined as the number of vOTUs with read counts above each threshold. (B) Species-level Shannon index. (C) Species-level Simpson index. Protocols A.1 and A.2 were excluded from the analysis due to the small number of samples (*n* = 2).

Viral enrichment (Protocol A) consistently recovered greater species-level richness than nonenriched protocols (A.0.1 and A.0.2). However, this increase was largely driven by a subset of highly abundant taxa (>20% relative abundance; >150 k reads; Figs 4 and S1). This skew toward high-abundance taxa is reflected in slightly reduced alpha diversity at low-abundance thresholds (<10%). Importantly, overall vOTU diversity at low read thresholds remains comparable across protocols, indicating that the observed differences primarily reflect enrichment of dominant taxa rather than a loss of low-abundance diversity (Figs 4 and S1–S3).

Library preparation strategies had moderate effects on overall richness and alpha diversity. Ligation-based libraries (A.3) exhibited slightly reduced vOTU richness across most abundance thresholds compared to tagmentation libraries (A), while showing comparable Shannon and Simpson diversity profiles (Fig. 4). Notably, differences in sequencing output strongly influence comparisons based on absolute read thresholds. Under these criteria, Protocol A.3 appears to underperform Protocol A, though this reflects sequencing depth rather than true methodological differences (Figs S1 and S3).

Nucleic acid extraction methods also had moderate effects on overall richness and alpha diversity. PureLink-based protocols (A.4 and A.4.1) yielded slightly higher vOTU richness and alpha diversity at lower abundance thresholds, while the QIAamp baseline (A) showed enrichment of a subset of high-abundance taxa (Fig. 4). Read-based thresholding also indicated that Protocol A underperformed the PureLink-based protocols (Figs S1 and S3). The addition of second-strand cDNA synthesis (A.4.1) did not result in appreciable differences relative to Protocol A.4. We did not assess richness and diversity metrics for Protocols A.1 (PEG precipitation) and A.2 (MDA amplification) because their small sample size (*n* = 2) limited reliable inference.

### Genome composition and structure of the vOTUs

**Figure 5:**
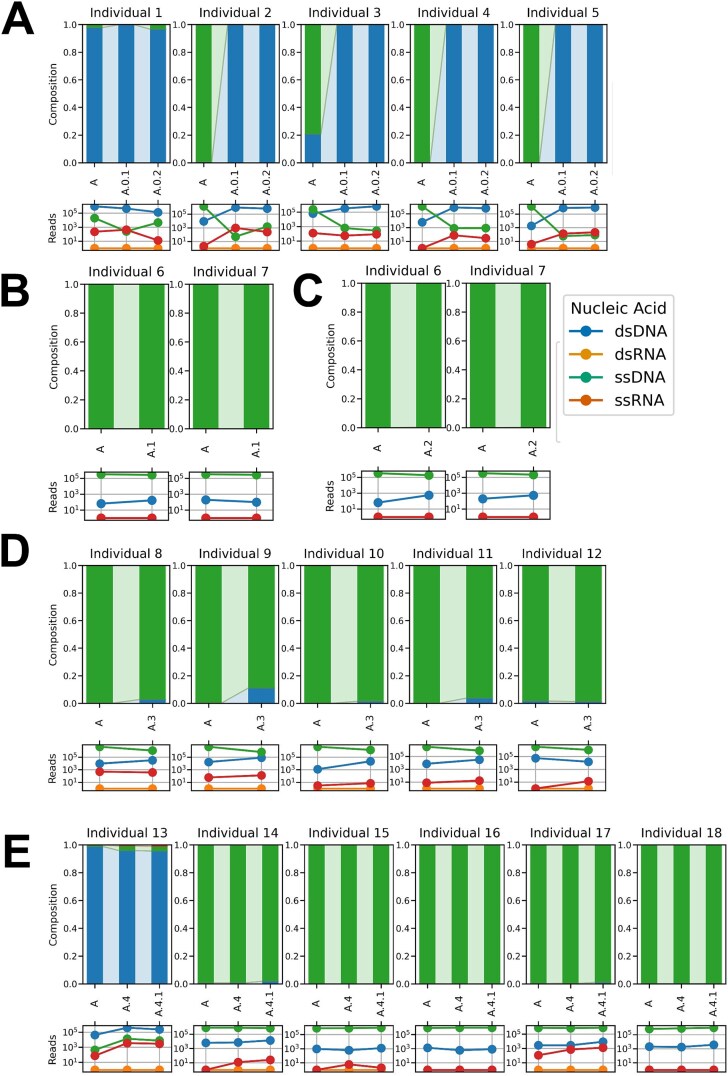
Diversity and composition of viral families by genome type. For A–E, the top figures represent the genome composition of individual samples, while the bottom ones show the reads of genome types (dsDNA, ssDNA, and ssRNA). (A) Viral enrichment, where Protocol A corresponds to viral precipitation by ultracentrifugation and Protocols A.0.1 and A.0.2 lack a viral enrichment step (nucleic acids were directly extracted using ZymoBIOMICS DNA/RNA Mini kit or AllPrep Power Viral DNA/RNA kit, respectively). (B) Viral enrichment strategy, where Protocol A corresponds to ultracentrifugation and Protocol A.1 to PEG-600-based precipitation. (C) Amplification strategy, where Protocol A corresponds to PCR-SISPA and Protocol A.2 to MDA. (D) Library preparation kit, where Protocol A corresponds to the DNA Prep (M) Tagmentation kit and Protocol A.3 to the TruSeq Nano DNA Library Prep kit. (E) DNA/RNA extraction, where Protocol A corresponds to the QIAamp Viral RNA Mini kit, Protocol A.4 to the PureLink Viral RNA/DNA Mini kit, and Protocol A.4.1 to the PureLink Viral RNA/DNA Mini kit followed by the cDNA second-strand synthesis.

We characterized genome type and structural traits by aggregating the proportion of reads mapping to vOTUs annotated as dsDNA, ssDNA, and ssRNA viruses for each protocol (Fig. 5). Across protocols, viral reads were predominantly assigned to ssDNA viruses (overall mean: 71.81%), which may be related to the amplification step as methods such as MDA and PCR-based approaches preferentially amplify ssDNA genomes, followed by dsDNA (28.13%) and ssRNA (0.06%) viruses. Genome type composition was more conserved within samples than between protocols, with greater variability observed between individuals. Consistent with these profiles, the recovered viral genomes were predominantly circular and nonenveloped, with genome sizes exceeding 200 kb for dsDNA viruses, and ranging between 4 and 8 kb for ssDNA viruses and between 10 and 18 kb for ssRNA viruses (Fig. S4).

**Figure 6:**
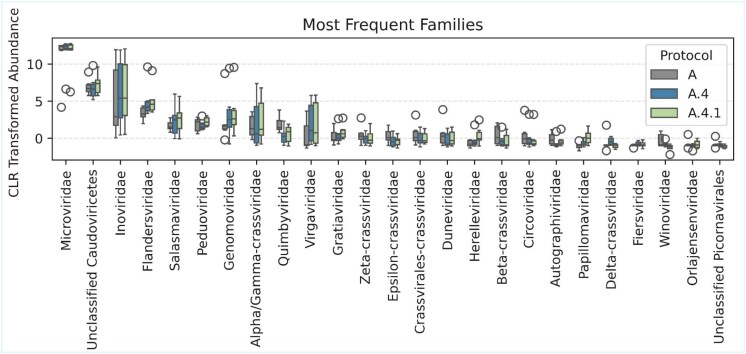
Most frequent viral families. Viral counts at the family level were compositionally transformed before applying the centered-log-ratio (CLR) to the data. The 25 families with the highest median CLR values are displayed. Protocol A corresponds to the QIAamp Viral RNA Mini kit, Protocol A.4 to the PureLink Viral RNA/DNA Mini kit, and Protocol A.4.1 to the PureLink Viral RNA/DNA Mini kit followed by the cDNA second-strand synthesis.

In the protocols without viral enrichment (A.0.1 and A.0.2), four of five samples showed a higher proportion of dsDNA viruses and a corresponding reduction in ssDNA viruses relative to the enriched baseline (Protocol A) (Fig. 5A). These differences coincided with changes in inferred structural features (Fig. S4), including a larger share of linear genomes, greater representation of viral particles >200 nm and an increased proportion of temperate phages in the nonenriched protocols. By contrast, choice of enrichment strategy (A.1: PEG vs A: ultracentrifugation), amplification strategy (A.2: MDA vs A: PCR-SISPA), and library chemistry (A.3: ligation vs A: tagmentation) did not produce major changes in viral genome type composition, with most protocols predominantly recovering ssDNA viruses across samples (Fig. 5B–D). Likewise, extraction chemistry (QIAamp in A vs PureLink in A.4 and A.4.1) yielded broadly comparable genome-type profiles, with ssDNA viruses dominated in five of six samples, while one donor consistently showed a higher relative abundance of dsDNA viruses across all three extraction workflows (Fig. 5E). Structural viral characteristics were also consistent across these comparisons (Fig. S4). Despite these overall consistencies, family-level composition revealed subtle protocol-dependent differences: comparison of the 25 most abundant viral families showed detectable shifts between Protocol A and the PureLink variants (A.4, A.4.1) (Fig. 6), suggesting that extraction chemistry can influence recovery of particular viral families, such as Flandersviridae and Genomoviridae, even when broad genome-type profiles remain stable.

To evaluate whether dominant viral groups masked protocol-dependent effects on less abundant viruses, we reanalyzed genome-type composition after removing reads assigned to the Microviridae family and the Caudoviricetes class, taxa that dominate many human fecal viromes [41, 50, 51]. After excluding these dominant groups, non-Microviridae ssDNA viruses still represented large fractions of the viral read pool across most samples and protocols (Fig. S5). However, differences in viral genome type composition persisted between Protocol A and nonenriched protocols (A.0.1 and A.0.2), with increased proportions of ssRNA viruses in the latter. By contrast, no relevant differences in genome type composition were observed between Protocol A and PEG-based purification (A.1), MDA-based amplification (A.2), ligation-based library preparation (A.3), or alternative nucleic acid extraction methods (A.4 and A.4.1), except for the increase in the relative proportions of ssRNA viruses in some of the individuals (Fig. S5). Structural and genomic characteristics remained consistent across these protocol comparisons (Fig. S6). Overall, the removal of dominant viral taxa showed that major protocol-dependent effects on genome type composition and structure are largely confined to protocols lacking viral enrichment; other methodological choices produced only subtle, individual-specific differences in the representation of less abundant viral groups.

## Discussion

Recent fecal virome research has made important strides in describing virome establishment and its perturbations in gastrointestinal disease, but methodological limitations remain a major barrier. In particular, RNA viruses are consistently underrepresented because common sample-preparation and sequencing protocols are biased toward DNA viruses [2, 52, 53]. Large-scale catalogs, including the Gut Virome Database [50], the Gut Phage Database [51], the Metagenomic Gut Virus catalog [54], and the UHGV catalog [41], have substantially expanded the repertoire of known gut-associated viruses, where most of the newly recovered diversity correspond to DNA viruses. These efforts have also exposed a critical problem: technical variability between studies often exceeds the biological variation of interest, confounding cross-study comparisons and meta-analyses. Motivated by these gaps, our study systematically assessed how methodological choices in virome metagenomics affect key outcomes, including viral recovery, contig quality, taxonomic assignment, diversity measures, and genome type composition, with the goal of identifying protocol choices that enhance viral detection while minimizing nonviral contamination.

We defined a reference protocol (A) by integrating and adapting previous published methods and technical recommendations [21–23, 32, 55] and used it as the baseline for all subsequent optimizations. Because recent extraction kits have been developed for broad microbial metagenomics, we tested whether direct total nucleic acid extraction could replace the more labor-intensive viral enrichment steps. We evaluated two commercial kits, ZymoBIOMICS DNA/RNA Miniprep and AllPrep PowerViral DNA/RNA, but both performed poorly relative to Protocol A, yielding a smaller fraction of reads mapping to vOTUs, fewer and shorter viral contigs predominantly classified as low-quality, and reduced viral richness. Together, these findings indicate that, despite convenience for general microbiome work, these kits are not well-suited for fecal virome metagenomic analyses when the goal is comprehensive viral recovery.

Because bacterial cells contribute far more nucleic acid than viruses, enrichment of viral particles is essential to enhance virome signal and sensitivity [22, 56, 57]. We therefore compared two common enrichment approaches, PEG-based precipitation and ultracentrifugation. PEG concentrates viral particles by excluding water molecules and is readily scalable [23, 58], while ultracentrifugation pellets viral particles efficiently [21]. Both methods delivered similar performance by several metrics: comparable percentages of reads mapped to vOTUs, similar numbers of high-quality contigs, and consistent genome-type distributions and structural features. However, PEG-based enrichment presented practical limitations, notably increased sample viscosity and occasional incomplete pellet recovery, consistent with previous reports [21]. These trade-offs suggest that while PEG is attractive for throughput and cost, ultracentrifugation may be preferable when maximal recovery consistency and ease of pellet handling are priorities.

The low abundance of viral nucleic acids in feces often requires an amplification step before library construction. However, when sufficient viral DNA is available, libraries can be prepared without amplification [14, 15] using methods such as Linker Amplified Shotgun Library (LASL) and the Adaptase–Linker Amplification(A-LA) [26, 59]. Eliminating the amplification step reduces representation bias and more accurately reflects relative viral abundances, though ssDNA viruses may be underrepresented as adapter ligation is generally more efficient for dsDNA [6, 30, 60]. In our comparisons, PCR-SISPA yielded a modestly higher proportion of viral reads than MDA, whereas MDA produced longer contigs and a greater number of complete genomes. MDA assemblies were notably enriched for circularized contigs and contigs exhibiting terminal repeats, consistent with the well-described Phi29 polymerase bias toward amplifying small circular ssDNA genomes (e.g. Microviridae) [40, 61]. This preferential amplification inflates the apparent abundance of viral families such as Microviridae [20, 30, 54, 61]. Microviridae has been reported as one of the most abundant viral groups in human fecal samples [50, 51] and its abundance has historically been linked to MDA-related artifacts [60]. In our study, reads mapping to Microviridae accounted for ~86% in the two MDA-amplified samples and ~97% in the PCR-SISPA-amplified samples. These observations underscore that amplification strategy remains a major source of methodological bias in virome studies: PCR-SISPA can introduce primer- and coverage-related biases [62], while MDA preferentially enriches circular ssDNA genomes. Consequently, disentangling true biological variation in viral community composition from amplification artifacts remains a central challenge, and results from amplified virome libraries should be interpreted with caution or validated using orthogonal, low-bias approaches when possible.

Library preparation remains one of the least standardized steps in virome metagenomics. While Illumina short-read sequencing remains the dominant platform, long-read technologies (Oxford Nanopore Technologies and PacBio) are increasingly used and are reshaping virome analyses [13, 63]. To our knowledge, this study represents the first direct comparison of commercial Illumina library preparation kits specifically for virome analysis. Both kits produced usable libraries, but they differed in important ways. TruSeq libraries yielded significantly fewer reads mapping to vOTUs and produced fewer medium-, high-quality, and complete viral contigs than DNA Prep libraries. In contrast, DNA Prep recovered higher viral richness and showed reduced evenness compared with TruSeq. Despite these differences, overall viral genome composition and structural features were consistent across kits and remained dominated by members of the Microviridae family. These findings align with prior bacterial metagenomics work showing that Nextera DNA Flex (now DNA Prep) reduces GC-related bias relative to Nextera XT, while TruSeq can provide more uniform coverage but may be biased in regions of extreme GC content [64]. Taken together, our results demonstrate that library-preparation chemistry influences viral recovery, contig quality, and diversity estimates. For studies that prioritize maximizing viral richness and genome completeness, DNA Prep appears preferable; however, kit choice should be guided by study goals, and cross-study comparisons must account for library-preparation differences.

When comparing nucleic acid extraction methods, we found that PureLink consistently outperformed Qiagen across all evaluated metrics, yielding a significantly higher fraction of reads mapping to vOTUs, more medium-, high-quality, and complete contigs, and higher richness and stable diversity across read-count thresholds. Despite these performance differences, the recovered viruses’ genomic and structural characteristics were comparable between kits. Adding a second-strand synthesis step provided no additional benefit and was therefore excluded from the final optimized protocol. Our findings align with those of Lewandowska *et al.* [31], who also observed reduced viral recovery, particularly for RNA viruses, using the Qiagen kit compared to PureLink and NucliSENS EasyMAG.

This study has several limitations. First, not all protocol modifications were tested on the same fecal samples, which may introduce sample-specific effects and limit direct head-to-head comparisons across every protocol combination. We deliberately used this design to control for individual-specific biases when assessing each modification, but it reduced the ability to generalize differences across protocols. Second, specific protocols—such as PEG precipitation and MDA amplification—were evaluated on only two samples, limiting statistical power and the generalizability of these findings. Third, although our wet-lab protocol was designed to recover both DNA and RNA viruses, downstream analysis relied primarily on the UHGV catalog, which predominantly contains dsDNA and ssDNA bacteriophages. As a result, RNA viruses were underrepresented in the final analyses, and any conclusion regarding RNA virus recovery should be treated cautiously. This shortcoming reflects current gaps in gut RNA-virus reference databases rather than the absence of RNA viruses in the samples and underscores the need for expanded RNA virus catalogs and tailored analytical pipelines to more accurately evaluate RNA virome recovery in future studies. Lastly, since our protocol relies on amplification, genome coverage does not reflect the relative abundance of viruses and is therefore more suitable for viral genome recovery than for quantitative virome ecology.

Furthermore, measures of viral diversity are highly sensitive to bioinformatic algorithm choices. Genome assembly can fail to recover low-abundance taxa, while read-mapping approaches are heavily dependent on the database used [65, 66]. Both approaches can introduce systematic biases that disproportionately affect low abundance or highly divergent taxa and can lead to distorted estimates of alpha diversity [67, 68]. To mitigate these effects, we combined assembly- and read-based methods and evaluated diversity across a series of abundance and read thresholds rather than relying on a single point estimate, which likewise can provide a misleading basis for comparative diversity analyses [69]. Nonetheless, samples may still appear less diverse simply because portions of the community are not recovered with our approach.

## Conclusion

Our optimized protocol for fecal virome analysis (Protocol A.4) combines bead-beating homogenization, viral enrichment—including low-speed centrifugation, 0.45 μm filtration, ultracentrifugation, and nuclease digestion—PureLink-based nucleic acid extraction, RNA retrotranscription, PCR-SISPA amplification, and tagmentation-based library preparation. This integrated workflow generates amplified viromes that consistently improved viral recovery, reduced nonviral contamination, increased viral diversity and genome completeness, and produced higher-quality contigs than the alternatives we tested. Key takeaways are the critical role of comprehensive viral enrichment, the superior performance of PureLink over QIAamp for nucleic acid extraction, the advantages of PCR-SISPA compared to MDA, and the benefits of tagmentation-based library construction. Together, these results establish a reproducible, high-yield protocol for human fecal virome studies and offer a practical baseline for future optimization and standardization efforts.

## Supplementary Material

Suppl_material_updated_ycag090_Figure_S1

Suppl_material_updated_ycag090_Figure_S2

Suppl_material_updated_ycag090_Figure_S3

Suppl_material_updated_ycag090_Figure_S4

Suppl_material_updated_ycag090_Figure_S5

Suppl_material_updated_ycag090_Figure_S6

Suppl_material_updated_ycag090_TableS1

Suppl_material_updated_ycag090_TableS2

Suppl_material_updated_ycag090

## Data Availability

Raw sequencing FASTQ files generated in this study are available in the BioProject repository under accession number PRJNA1250239. All code and scripts used for bioinformatic processing, taxonomic assignment, assembly, richness and diversity analysis, and statistical analyses are publicly available at: https://github.com/rdoughty10/GutVirome.

## References

[1] Bhagchandani T, Nikita VA, Tandon R. Exploring the human virome: composition, dynamics, and implications for health and disease. Curr Microbiol 2023;81:16. 10.1007/s00284-023-03537-0 38006423

[2] Cao Z, Sugimura N, Burgermeister E et al. The gut virome: a new microbiome component in health and disease. EBioMedicine 2022;81:104113. 10.1016/j.ebiom.2022.104113 35753153 PMC9240800

[3] Liang G, Bushman FD. The human virome: assembly, composition and host interactions. Nat Rev Microbiol 2021;19:514–27. 10.1038/s41579-021-00536-5 33785903 PMC8008777

[4] Jansen D, Matthijnssens J. The emerging role of the gut virome in health and inflammatory bowel disease: challenges, covariates and a viral imbalance. Viruses 2023;15:173. 10.3390/v15010173 36680214 PMC9861652

[5] Sutton TDS, Clooney AG, Ryan FJ et al. Choice of assembly software has a critical impact on virome characterisation. Microbiome 2019;7:12. 10.1186/s40168-019-0626-5 30691529 PMC6350398

[6] Khan Mirzaei M, Xue J, Costa R et al. Challenges of studying the human virome - relevant emerging technologies. Trends Microbiol 2021;29:171–81. 10.1016/j.tim.2020.05.021 32622559

[7] Krishnamurthy SR, Wang D. Origins and challenges of viral dark matter. Virus Res 2017;239:136–42. 10.1016/j.virusres.2017.02.002 28192164

[8] García-López R, Pérez-Brocal V, Moya A. Beyond cells - the virome in the human holobiont. Microb Cell 2019;6:373–96. 10.15698/mic2019.09.689 31528630 PMC6717880

[9] Santiago-Rodriguez TM, Hollister EB. Unraveling the viral dark matter through viral metagenomics. Front Immunol 2022;13:1005107. 10.3389/fimmu.2022.1005107 36189246 PMC9523745

[10] Roux S, Coclet C. Viromics approaches for the study of viral diversity and ecology in microbiomes. Nat Rev Genet 2026;27:32–46. 10.1038/s41576-025-00871-w 40691354

[11] Angly FE, Felts B, Breitbart M et al. The marine viromes of four oceanic regions. PLoS Biol 2006;4:e368. 10.1371/journal.pbio.0040368 17090214 PMC1634881

[12] Wylie KM, Weinstock GM, Storch GA. Emerging view of the human virome. Transl Res 2012;160:283–90. 10.1016/j.trsl.2012.03.006 22683423 PMC3701101

[13] Xu Y, Lewandowski K, Lumley S et al. Detection of viral pathogens with multiplex nanopore MinION sequencing: Be careful with cross-talk. Front Microbiol 2018;9:2225. 10.3389/fmicb.2018.02225 30283430 PMC6156371

[14] Shkoporov AN, Clooney AG, Sutton TDS et al. The human gut virome is highly diverse, stable, and individual specific. Cell Host Microbe 2019;26:527–541.e5. 10.1016/j.chom.2019.09.009 31600503

[15] Stockdale SR, Shkoporov AN, Khokhlova EV et al. Interpersonal variability of the human gut virome confounds disease signal detection in IBD. Commun Biol 2023;6:221. 10.1038/s42003-023-04592-w 36841913 PMC9968284

[16] Hillary LS, Knotts TA, Adams SH et al. DNA extraction and virome processing methods strongly influence recovered human gut viral community characteristics. bioRxivorg bioRxiv 2025. 10.1101/2025.11.25.690293

[17] Zuppi M, Hendrickson HL, O’Sullivan JM et al. Phages in the gut ecosystem. Front Cell Infect Microbiol 2021;11:822562. 10.3389/fcimb.2021.822562 35059329 PMC8764184

[18] Briese T, Kapoor A, Mishra N et al. Virome capture sequencing enables sensitive viral diagnosis and comprehensive virome analysis. MBio 2015;6:e01491-15. 10.1128/mbio.01491-15 PMC4611031 26396248

[19] Wylie TN, Wylie KM, Herter BN et al. Enhanced virome sequencing using targeted sequence capture. Genome Res 2015;25:1910–20. 10.1101/gr.191049.115 26395152 PMC4665012

[20] Callanan J, Stockdale SR, Shkoporov A et al. Biases in viral metagenomics-based detection, cataloguing and quantification of bacteriophage genomes in human faeces, a review. Microorganisms 2021;9:524. 10.3390/microorganisms9030524 33806607 PMC8000950

[21] Kleiner M, Hooper LV, Duerkop BA. Evaluation of methods to purify virus-like particles for metagenomic sequencing of intestinal viromes. BMC Genomics 2015;16:7. 10.1186/s12864-014-1207-4 25608871 PMC4308010

[22] Conceição-Neto N, Zeller M, Lefrère H et al. Modular approach to customise sample preparation procedures for viral metagenomics: a reproducible protocol for virome analysis. Sci Rep 2015;5:16532. 10.1038/srep16532 26559140 PMC4642273

[23] Shkoporov AN, Ryan FJ, Draper LA et al. Reproducible protocols for metagenomic analysis of human faecal phageomes. Microbiome 2018;6:68. 10.1186/s40168-018-0446-z 29631623 PMC5892011

[24] Zhang D, Lou X, Yan H et al. Metagenomic analysis of viral nucleic acid extraction methods in respiratory clinical samples. BMC Genomics 2018;19:773. 10.1186/s12864-018-5152-5 30359242 PMC6202819

[25] Sabatier M, Bal A, Destras G et al. Comparison of nucleic acid extraction methods for a viral metagenomics analysis of respiratory viruses. Microorganisms 2020;8:1539. 10.3390/microorganisms8101539 33036303 PMC7601816

[26] Roux S, Solonenko NE, Dang VT et al. Towards quantitative viromics for both double-stranded and single-stranded DNA viruses. PeerJ 2016;4:e2777. 10.7717/peerj.2777 28003936 PMC5168678

[27] Kim D, Song L, Breitwieser FP et al. Centrifuge: rapid and sensitive classification of metagenomic sequences. Genome Res 2016;26:1721–9. 10.1101/gr.210641.116 27852649 PMC5131823

[28] Corinaldesi C, Tangherlini M, Dell’Anno A. From virus isolation to metagenome generation for investigating viral diversity in deep-sea sediments. Sci Rep 2017;7:8355. 10.1038/s41598-017-08783-4 28827715 PMC5566222

[29] Thornton M, Eder G, Amman F et al. Comparative wastewater virome analysis with different enrichment methods. Water Res 2025;285:123985. 10.1016/j.watres.2025.123985 40570497

[30] Parras-Moltó M, Rodríguez-Galet A, Suárez-Rodríguez P et al. Evaluation of bias induced by viral enrichment and random amplification protocols in metagenomic surveys of saliva DNA viruses. Microbiome 2018;6:119. 10.1186/s40168-018-0507-3 29954453 PMC6022446

[31] Lewandowska DW, Zagordi O, Geissberger F-D et al. Optimization and validation of sample preparation for metagenomic sequencing of viruses in clinical samples. Microbiome 2017;5:94. 10.1186/s40168-017-0317-z 28789678 PMC5549297

[32] L Kramná, O Cinek. Virome sequencing of stool samples. Methods Mol Biol 2018;1838: 59–83, New York, NY, Springer New York, 10.1007/978-1-4939-8682-8_6. 30128990

[33] Chen S, Zhou Y, Chen Y et al. Fastp: an ultra-fast all-in-one FASTQ preprocessor. Bioinformatics 2018;34:i884–90. 10.1093/bioinformatics/bty560 30423086 PMC6129281

[34] Langmead B, Salzberg SL. Fast gapped-read alignment with bowtie 2. Nat Methods 2012;9:357–9. 10.1038/nmeth.1923 22388286 PMC3322381

[35] Babraham Bioinformatics - FastQC a Quality Control Tool for High Throughput Sequence Data http://www.bioinformatics.babraham.ac.uk/projects/fastqc/. Date accessed 25 March 2025.

[36] Ewels P, Magnusson M, Lundin S et al. MultiQC: summarize analysis results for multiple tools and samples in a single report. Bioinformatics 2016;32:3047–8. 10.1093/bioinformatics/btw354 27312411 PMC5039924

[37] Zolfo M, Pinto F, Asnicar F et al. Detecting contamination in viromes using ViromeQC. Nat Biotechnol 2019;37:1408–12. 10.1038/s41587-019-0334-5 31748692

[38] Li D, Liu C-M, Luo R et al. MEGAHIT: an ultra-fast single-node solution for large and complex metagenomics assembly via succinct de Bruijn graph. Bioinformatics 2015;31:1674–6. 10.1093/10.1093/bioinformatics/btv033 25609793

[39] Camargo AP, Roux S, Schulz F et al. Identification of mobile genetic elements with geNomad. Nat Biotechnol 2024;42:1303–12. 10.1038/s41587-023-01953-y 37735266 PMC11324519

[40] Nayfach S, Camargo AP, Schulz F et al. CheckV assesses the quality and completeness of metagenome-assembled viral genomes. Nat Biotechnol 2021;39:578–85. 10.1038/s41587-020-00774-7 33349699 PMC8116208

[41] Camargo AP, Baltoumas FA, Ndela EO et al. A genomic atlas of the human gut virome elucidates genetic factors shaping host interactions. bioRxivorg 2025. 10.1101/2025.11.01.686033

[42] Camacho C, Coulouris G, Avagyan V et al. BLAST+: architecture and applications. BMC Bioinformatics 2009;10:421. 10.1186/1471-2105-10-421 20003500 PMC2803857

[43] Li H, Handsaker B, Wysoker A et al. The sequence alignment/map format and SAMtools. Bioinformatics 2009;25:2078–9. 10.1093/bioinformatics/btp352 19505943 PMC2723002

[44] Lefkowitz EJ, Dempsey DM, Hendrickson RC et al. Virus taxonomy: the database of the International Committee on Taxonomy of Viruses (ICTV). Nucleic Acids Res 2018;46:D708–17. 10.1093/nar/gkx932 29040670 PMC5753373

[45] Hulo C, de Castro E, Masson P et al. ViralZone: a knowledge resource to understand virus diversity. Nucleic Acids Res 2011;39:D576–82. 10.1093/nar/gkq901 20947564 PMC3013774

[46] Shannon CE . A mathematical theory of communication. Bell Syst Tech J 1948;27:379–423. 10.1002/j.1538-7305.1948.tb01338.x

[47] Simpson EH . Measurement of diversity. Nature 1949;163:688–8. 10.1038/163688a0

[48] Hristova K, Wimley WC. Determining the statistical significance of the difference between arbitrary curves: a spreadsheet method. PloS One 2023;18:e0289619. 10.1371/journal.pone.0289619 37906570 PMC10617697

[49] Lin H, Peddada SD. Analysis of microbial compositions: a review of normalization and differential abundance analysis. NPJ Biofilms Microbiomes 2020;6:60. 10.1038/s41522-020-00160-w 33268781 PMC7710733

[50] Gregory AC, Zablocki O, Zayed AA et al. The gut Virome database reveals age-dependent patterns of virome diversity in the human gut. Cell Host Microbe 2020;28:724–740.e8. 10.1016/j.chom.2020.08.003 32841606 PMC7443397

[51] Camarillo-Guerrero LF, Almeida A, Rangel-Pineros G et al. Massive expansion of human gut bacteriophage diversity. Cell 2021;184:1098–1109.e9. 10.1016/j.cell.2021.01.029 33606979 PMC7895897

[52] Chen F, Li S, Guo R et al. Meta-analysis of fecal viromes demonstrates high diagnostic potential of the gut viral signatures for colorectal cancer and adenoma risk assessment. J Adv Res 2023;49:103–14. 10.1016/j.jare.2022.09.012 36198381 PMC10334131

[53] Pargin E, Roach MJ, Skye A et al. The human gut virome: composition, colonization, interactions, and impacts on human health. Front Microbiol 2023;14:963173. 10.3389/fmicb.2023.963173 37293229 PMC10244655

[54] Nayfach S, Páez-Espino D, Call L et al. Metagenomic compendium of 189,680 DNA viruses from the human gut microbiome. Nat Microbiol 2021;6:960–70. 10.1038/s41564-021-00928-6 34168315 PMC8241571

[55] Hall RJ, Wang J, Todd AK et al. Evaluation of rapid and simple techniques for the enrichment of viruses prior to metagenomic virus discovery. J Virol Methods 2014;195:194–204. 10.1016/j.jviromet.2013.08.035 24036074 PMC7113663

[56] Bassi C, Guerriero P, Pierantoni M et al. Novel virus identification through metagenomics: a systematic review. Life (Basel) 2022;12:2048. 10.3390/life12122048 36556413 PMC9784588

[57] Wang G, Li S, Yan Q et al. Optimization and evaluation of viral metagenomic amplification and sequencing procedures toward a genome-level resolution of the human fecal DNA virome. J Adv Res 2023;48:75–86. 10.1016/j.jare.2022.08.011 35995413 PMC10248800

[58] Hoyles L, McCartney AL, Neve H et al. Characterization of virus-like particles associated with the human faecal and caecal microbiota. Res Microbiol 2014;165:803–12. 10.1016/j.resmic.2014.10.006 25463385

[59] Kim K-H, Bae J-W. Amplification methods bias metagenomic libraries of uncultured single-stranded and double-stranded DNA viruses. Appl Environ Microbiol 2011;77:7663–8. 10.1128/AEM.00289-11 21926223 PMC3209148

[60] Reyes A, Semenkovich NP, Whiteson K et al. Going viral: next-generation sequencing applied to phage populations in the human gut. Nat Rev Microbiol 2012;10:607–17. 10.1038/nrmicro2853 22864264 PMC3596094

[61] Tisza MJ, Pastrana DV, Welch NL et al. Discovery of several thousand highly diverse circular DNA viruses. Elife 2020;9:e5. 10.7554/eLife.51971 PMC7000223 32014111

[62] Karlsson OE, Belák S, Granberg F. The effect of preprocessing by sequence-independent, single-primer amplification (SISPA) on metagenomic detection of viruses. Biosecur Bioterror 2013;11:S227–34. 10.1089/bsp.2013.0008 23971810

[63] Yahara K, Suzuki M, Hirabayashi A et al. Long-read metagenomics using PromethION uncovers oral bacteriophages and their interaction with host bacteria. Nat Commun 2021;12:27. 10.1038/s41467-020-20199-9 33397904 PMC7782811

[64] Sato MP, Ogura Y, Nakamura K et al. Comparison of the sequencing bias of currently available library preparation kits for Illumina sequencing of bacterial genomes and metagenomes. DNA Res 2019;26:391–8. 10.1093/dnares/dsz017 31364694 PMC6796507

[65] Luan T, Cepeda-Espinoza VP, Liu B et al. Reference-guided assembly of metagenomes with MetaCompass. Cell Rep Methods 2025;5:101186. 10.1016/j.crmeth.2025.101186 41015028 PMC12570322

[66] Nasko DJ, Koren S, Phillippy AM et al. RefSeq database growth influences the accuracy of k-mer-based lowest common ancestor species identification. Genome Biol 2018;19:165. 10.1186/s13059-018-1554-6 30373669 PMC6206640

[67] Zhao C, Shi ZJ, Pollard KS. Pitfalls of genotyping microbial communities with rapidly growing genome collections. Cell Syst 2023;14:160–176.e3. 10.1016/j.cels.2022.12.007 36657438 PMC9957970

[68] Smith RH, Glendinning L, Walker AW et al. Investigating the impact of database choice on the accuracy of metagenomic read classification for the rumen microbiome. Anim Microbiome 2022;4:57. 10.1186/s42523-022-00207-7 36401288 PMC9673341

[69] Willis AD . Rarefaction, alpha diversity, and statistics. Front Microbiol 2019;10:2407. 10.3389/fmicb.2019.02407 31708888 PMC6819366

