# Supplementary material for: A comparison of methods for the optimal recovery of the human fecal virome
Source: ISME Commun. 2026 Apr 11;6(1):ycag090. doi: 10.1093/ismeco/ycag090 (PMC13155102; doi:10.1093/ismeco/ycag090)
Supplement: Suppl_material_updated_ycag090_Figure_S2 [file suppl_material_updated_ycag090_figure_s2.pdf]

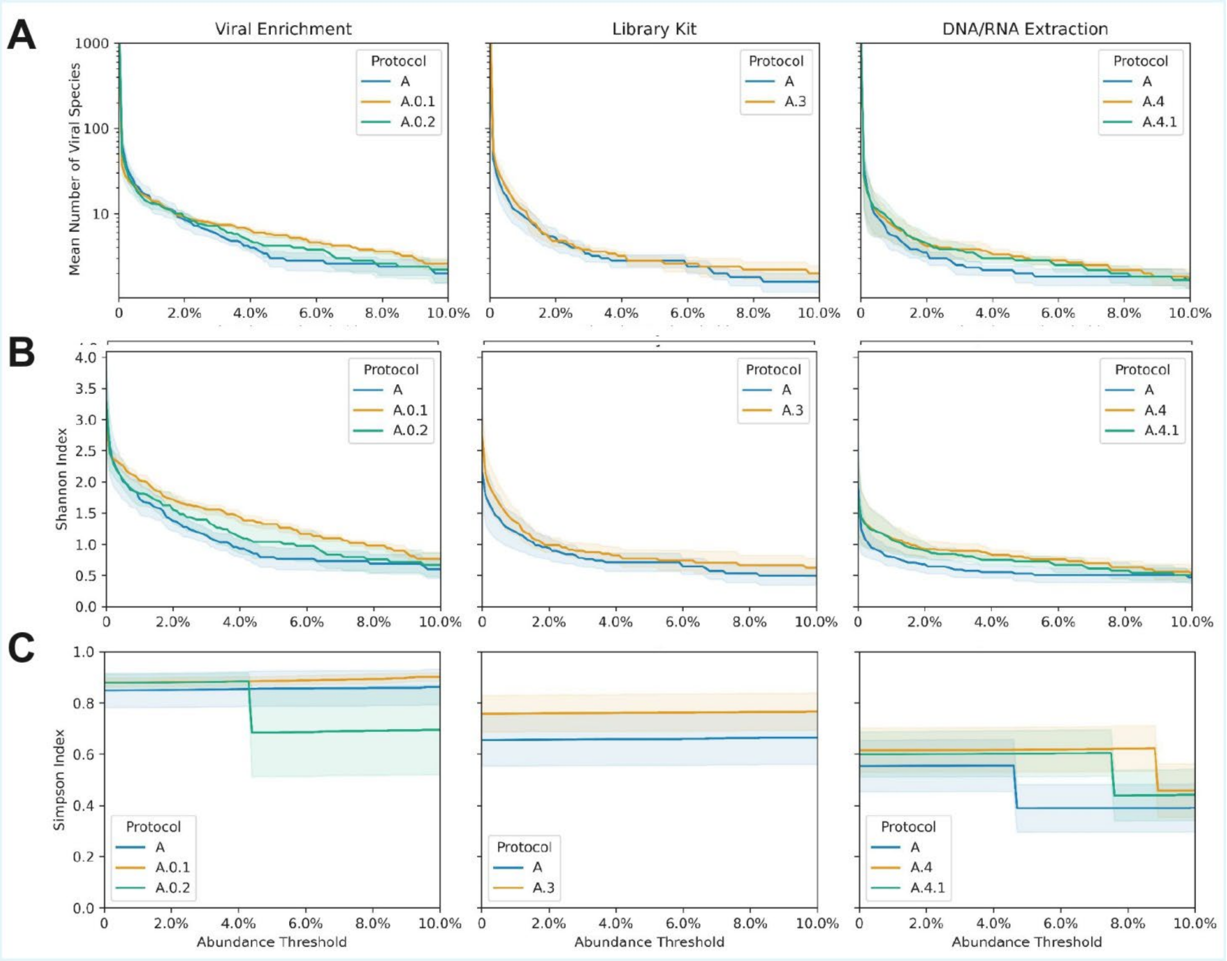

Figure S2: Low abundance diversity. Richness and diversity metrics of viral taxonomic assignments across protocols. Metrics were calculated using vOTUs, treated as species-level units, and calculated at increasing abundance thresholds, disregarding any taxa below the required abundance. Cutoffs from 0-10% with step sizes of 0.1% were calculated. A) Species-level richness, defined as the number of vOTUs with read counts above each threshold. B) Species-level Shannon index. C) Species-level Simpson index. Protocols A.1 and A.2 were excluded from the analysis due to the small number of samples (n=2).
