# Supplementary material for: A comparison of methods for the optimal recovery of the human fecal virome
Source: ISME Commun. 2026 Apr 11;6(1):ycag090. doi: 10.1093/ismeco/ycag090 (PMC13155102; doi:10.1093/ismeco/ycag090)
Supplement: Suppl_material_updated_ycag090_Figure_S4 [file suppl_material_updated_ycag090_figure_s4.pdf]

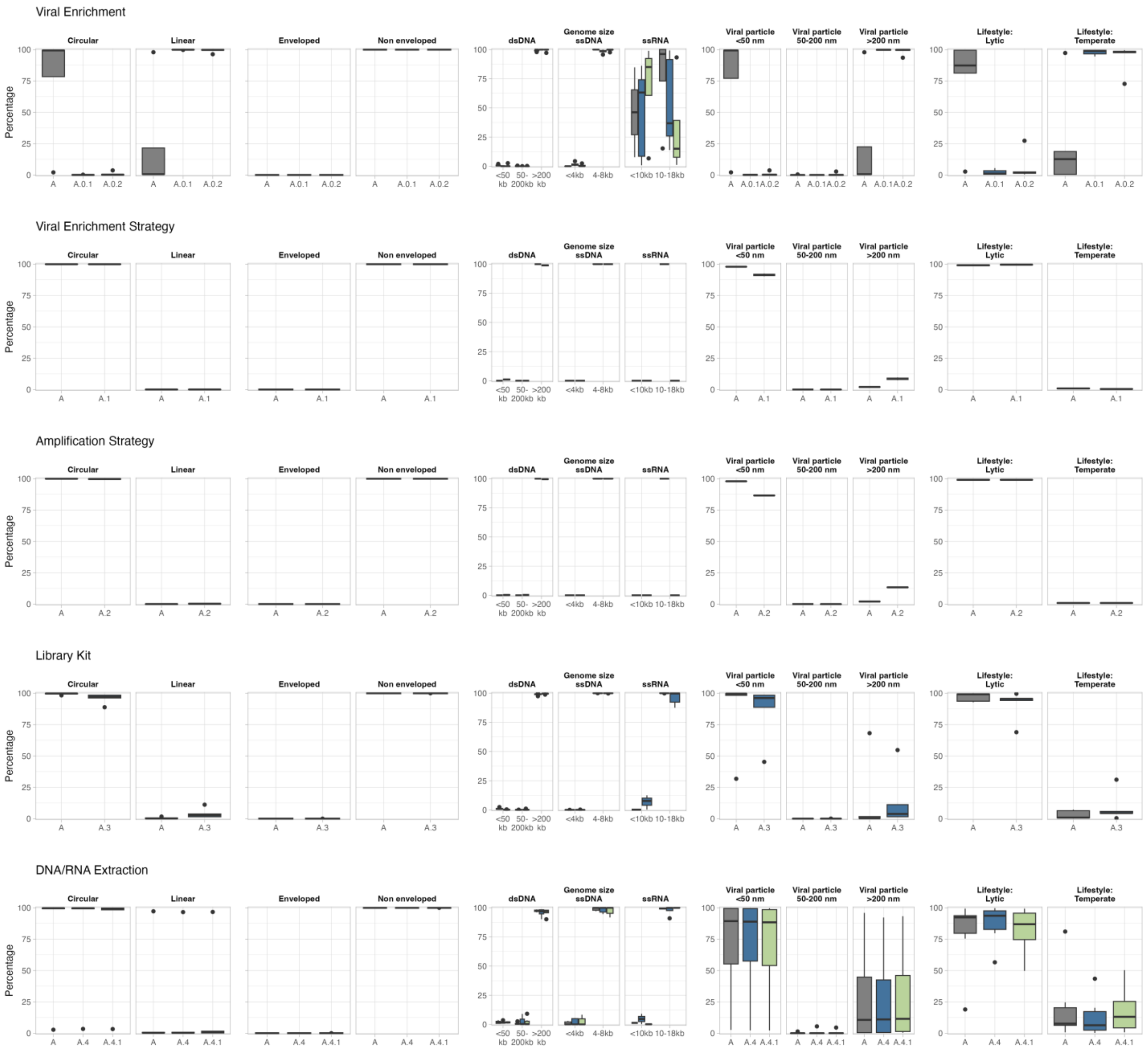

Figure S4: Structural and genomic characteristics of the viral families identified by each protocol. Boxplots for each protocol showing the characteristics of viruses: circular or linear genome, enveloped or non-enveloped structure, genome size, viral particle size (<50, 50-200 and >200 nm), and the proportion of lytic and temperate phages. The central line represents the median, box limits indicate Q1 and Q3, and whiskers extend to the lowest and highest non-outlier values (Q1-1.5 IQR and Q3+1.5 IQR).
