# Supplementary material for: A comparison of methods for the optimal recovery of the human fecal virome
Source: ISME Commun. 2026 Apr 11;6(1):ycag090. doi: 10.1093/ismeco/ycag090 (PMC13155102; doi:10.1093/ismeco/ycag090)
Supplement: Suppl_material_updated_ycag090_Figure_S5 [file suppl_material_updated_ycag090_figure_s5.pdf]

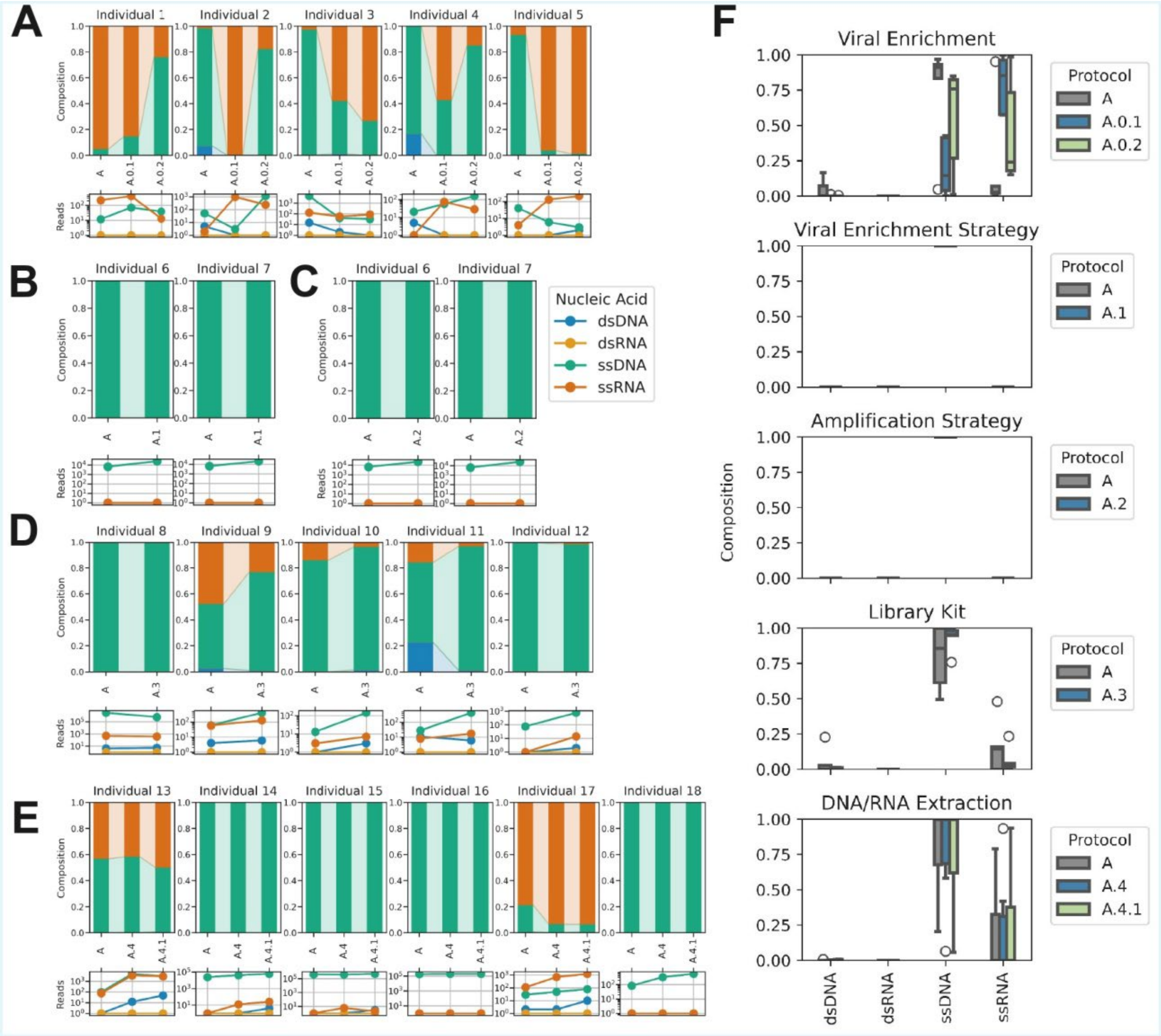

Figure S5: Composition of viruses by genome type for the protocols tested after excluding the *Microviridae* family and *Caudoviricetes* class. For A-E, the top figures represent the genome composition of individual samples, while the bottom one shows the reads of genome types (dsDNA, ssDNA and ssRNA). A) Viral enrichment, where protocol A corresponds to viral precipitation by ultracentrifugation and protocols A.0.1 and A.0.2 lack a viral enrichment step (nucleic acids were directly extracted using ZymoBIOMICS DNA/RNA Mini kit or AllPrep Power Viral DNA/RNA kit, respectively). B) Viral enrichment strategy, where protocol A corresponds to ultracentrifugation and protocol A.1 to PEG-600-based precipitation. C) Amplification strategy, where protocol A corresponds to PCR-SISPA and protocol A.2 to MDA. D) Library preparation kit, where protocol A corresponds to the DNA Prep (M) Tagmentation kit and protocol A.3 to the TruSeq Nano DNA Library Prep kit. E) DNA/RNA extraction, where protocol A corresponds to the QIAamp Viral RNA Mini kit, protocol A.4 to the PureLink Viral RNA/DNA Mini kit, and protocol A.4.1 to PureLink Viral RNA/DNA Mini kit followed by the cDNA second-strand synthesis. F) Boxplots showing the genome composition for the protocols. The central line represents the median, box limits indicate the Q1 and Q3 quartiles, and whiskers extend to Q1-1.5 IQR and Q3+1.5 IQR, marking the lowest and highest non-outlier values.
