# Supplementary material for: A comparison of methods for the optimal recovery of the human fecal virome
Source: ISME Commun. 2026 Apr 11;6(1):ycag090. doi: 10.1093/ismeco/ycag090 (PMC13155102; doi:10.1093/ismeco/ycag090)
Supplement: Suppl_material_updated_ycag090_TableS1 [file suppl_material_updated_ycag090_tables1.pdf]

		Fastp	Human Read Removal	
**file**	**Raw Reads (R1)**	**Reads After**	**Reads Removed**	**Reads After**
9092-U-virome_S11_L001_R1.fastq	1664460	1417212	995	1416217
9092-1Q-virome_S3_L001_R1.fastq	1742277	1419592	3481	1416111
9092-2Z-virome_S1_L001_R1.fastq	1456005	1181383	175	1181208
2820-U-virome_S14_L001_R1.fastq	1691399	1447463	2336	1445127
2820-2Q-virome_S8_L001_R1.fastq	1460188	1261848	96	1261752
2820-2Z-virome_S2_L001_R1.fastq	1697036	1510265	76	1510189
9324-U-virome_S12_L001_R1.fastq	1600505	1213434	17666	1195768
9324-2Q-virome_S5_L001_R1.fastq	1542093	1396271	91	1396180
9324-2Z-virome_S4_L001_R1.fastq	1375155	1191969	1565	1190404
0168-U-virome_S13_L001_R1.fastq	1538372	1329715	3111	1326604
0168-1Q-virome_S7_L001_R1.fastq	1499517	1297634	1343	1296291
0168-1Z-virome_S6_L001_R1.fastq	1862747	1591820	2930	1588890
8466-U-virome_S17_L001_R1.fastq	1555191	1356565	322	1356243
8466-1Q-virome_S10_L001_R1.fastq	1673012	1367067	255	1366812
8466-2Z-virome_S9_L001_R1.fastq	1410253	1237346	767	1236579
SAC1-viromeR_S55_L001_R1.fastq	365571	331725	12	331713
SAC2-viromeR_S56_L001_R1.fastq	368602	337085	11	337074
SBC1-virome_R1.fastq	307751	283223	112	283111
SBC2-virome_R1.fastq	321036	293170	18	293152
SAD1-viromeR_S61_L001_R1.fastq	227262	213851	4	213847
SAD2-virome_R1.fastq	260881	244483	11	244472
S1-5393-VIROME_S1_L001_R1.fastq	5131783	4617437	3105	4614332
S1-5393-viromeTruSeq.fastq	1654933	1624596	1002	1623594
S3-9504-VIROME_S3_L001_R1.fastq	4781559	4434669	2810	4431859
S3-9504-viromeTruSeq.fastq	1217574	1021570	685	1020885
S4-1876-VIROME_S4_L001_R1.fastq	4913300	4540936	316	4540620
S4-1876-viromeTruSeq.fastq	1694898	1639087	112	1638975
14-2820AC-VIROME_S5_L001_R1.fastq	3608775	3218914	3015	3215899

14-2820AC-viromeTruSeq.fastq	1390669	1333453	335	1333118
15-8466AC-VIROME_S6_L001_R1.fastq	3811556	3541076	423	3540653
15-8466AC-viromeTruSeq.fastq	1350120	1321047	113	1320934
P1-2635-I-virome_R1.fastq	897795	739396	39988	699408
P1-2635-Q-virome_R1.fastq	1020060	856378	3949	852429
P1-2635-Ids-virome_R1.fastq	651458	489337	48254	441083
P2-6080-I-virome_R1.fastq	919576	834110	2049	832061
P2-6080-Q-virome_R1.fastq	1018526	904308	414	903894
P2-6080-Ids-virome_R1.fastq	830553	744404	4126	740278
P3-5566-I-virome_R1.fastq	869113	791244	924	790320
P3-5566-Q-virome_R1.fastq	895461	804130	770	803360
P3-5566-Ids-virome_R1.fastq	1018301	916744	2171	914573
P4-1728-I-virome_R1.fastq	999725	908923	786	908137
P4-1728-Q-virome_R1.fastq	969928	880027	950	879077
P4-1728-Ids-virome_R1.fastq	983342	906062	1385	904677
P5-8986-I-virome_R1.fastq	758798	693210	3668	689542
P5-8986-Q-virome_R1.fastq	891602	821949	2757	819192
P5-8986-Ids-virome_R1.fastq	903287	815061	11596	803465
P6-8992-I-virome_R1.fastq	873992	789156	787	788369
P6-8992-Q-virome_R1.fastq	746119	681266	599	680667
P6-8992-Ids-virome_R1.fastq	1103322	987145	1921	985224

file	format	type	Reads After Fastp	sum_len	min_len	avg_len	max_len
9092-U-virome_S11_L001_R1.fastq	FASTQ	DNA	1417212	150912234	50	106.5	151
9092-U-virome_S11_L001_R2.fastq	FASTQ	DNA	1417212	150612398	50	106.3	151
9092-1Q-virome_S3_L001_R1.fastq	FASTQ	DNA	1419592	135065033	50	95.1	151
9092-1Q-virome_S3_L001_R2.fastq	FASTQ	DNA	1419592	134676676	50	94.9	151
9092-2Z-virome_S1_L001_R1.fastq	FASTQ	DNA	1181383	117247142	50	99.2	151
9092-2Z-virome_S1_L001_R2.fastq	FASTQ	DNA	1181383	116990968	50	99	151
2820-U-virome_S14_L001_R1.fastq	FASTQ	DNA	1447463	158570239	50	109.6	151
2820-U-virome_S14_L001_R2.fastq	FASTQ	DNA	1447463	158298344	50	109.4	151
2820-2Q-virome_S8_L001_R1.fastq	FASTQ	DNA	1261848	124280606	50	98.5	151
2820-2Q-virome_S8_L001_R2.fastq	FASTQ	DNA	1261848	123946963	50	98.2	151
2820-2Z-virome_S2_L001_R1.fastq	FASTQ	DNA	1510265	165383692	50	109.5	151
2820-2Z-virome_S2_L001_R2.fastq	FASTQ	DNA	1510265	165161416	50	109.4	151
9324-U-virome_S12_L001_R1.fastq	FASTQ	DNA	1213434	121680025	50	100.3	151
9324-U-virome_S12_L001_R2.fastq	FASTQ	DNA	1213434	121315226	50	100	151
9324-2Q-virome_S5_L001_R1.fastq	FASTQ	DNA	1396271	155501595	50	111.4	151
9324-2Q-virome_S5_L001_R2.fastq	FASTQ	DNA	1396271	155197498	50	111.2	151
9324-2Z-virome_S4_L001_R1.fastq	FASTQ	DNA	1191969	127427880	50	106.9	151
9324-2Z-virome_S4_L001_R2.fastq	FASTQ	DNA	1191969	127112831	50	106.6	151
0168-U-virome_S13_L001_R1.fastq	FASTQ	DNA	1329715	145641658	50	109.5	151
0168-U-virome_S13_L001_R2.fastq	FASTQ	DNA	1329715	145356512	50	109.3	151
0168-1Q-virome_S7_L001_R1.fastq	FASTQ	DNA	1297634	140486614	50	108.3	151
0168-1Q-virome_S7_L001_R2.fastq	FASTQ	DNA	1297634	140179597	50	108	151
0168-1Z-virome_S6_L001_R1.fastq	FASTQ	DNA	1591820	163870803	50	102.9	151
0168-1Z-virome_S6_L001_R2.fastq	FASTQ	DNA	1591820	163539449	50	102.7	151
8466-U-virome_S17_L001_R1.fastq	FASTQ	DNA	1356565	152210113	50	112.2	151
8466-U-virome_S17_L001_R2.fastq	FASTQ	DNA	1356565	151986613	50	112	151
8466-1Q-virome_S10_L001_R1.fastq	FASTQ	DNA	1367067	132809156	50	97.1	151
8466-1Q-virome_S10_L001_R2.fastq	FASTQ	DNA	1367067	132435012	50	96.9	151
8466-2Z-virome_S9_L001_R1.fastq	FASTQ	DNA	1237346	131272792	50	106.1	151

8466-2Z-virome_S9_L001_R2.fastq	FASTQ	DNA	1237346	130968555	50	105.8	151
SAC1-viromeR_S55_L001_R1.fastq	FASTQ	DNA	331725	39031581	50	117.7	151
SAC1-viromeR_S55_L001_R2.fastq	FASTQ	DNA	331725	38974631	50	117.5	151
SAC2-viromeR_S56_L001_R1.fastq	FASTQ	DNA	337085	40042025	50	118.8	151
SAC2-viromeR_S56_L001_R2.fastq	FASTQ	DNA	337085	39984546	50	118.6	151
SBC1-virome_R1.fastq	FASTQ	DNA	283223	33736104	50	119.1	151
SBC1-virome_R2.fastq	FASTQ	DNA	283223	33692234	50	119	151
SBC2-virome_R1.fastq	FASTQ	DNA	293170	34506971	50	117.7	151
SBC2-virome_R2.fastq	FASTQ	DNA	293170	34461852	50	117.5	151
SAD1-viromeR_S61_L001_R1.fastq	FASTQ	DNA	213851	27269445	50	127.5	151
SAD1-viromeR_S61_L001_R2.fastq	FASTQ	DNA	213851	27236344	50	127.4	151
SAD2-virome_R1.fastq	FASTQ	DNA	244483	31470425	50	128.7	151
SAD2-virome_R2.fastq	FASTQ	DNA	244483	31440229	50	128.6	151
S1-5393-VIROME_S1_L001_R1.fastq	FASTQ	DNA	4617437	539934391	50	116.9	151
S1-5393-VIROME_S1_L001_R2.fastq	FASTQ	DNA	4617437	539152616	50	116.8	151
S1-5393-viromeTruSeq.fastq	FASTQ	DNA	1624596	237353678	50	146.1	151
S3-9504-VIROME_S3_L001_R1.fastq	FASTQ	DNA	4434669	539628515	50	121.7	151
S3-9504-VIROME_S3_L001_R2.fastq	FASTQ	DNA	4434669	539073611	50	121.6	151
S3-9504-viromeTruSeq.fastq	FASTQ	DNA	1021570	132551922	50	129.8	151
S4-1876-VIROME_S4_L001_R1.fastq	FASTQ	DNA	4540936	557296874	50	122.7	151
S4-1876-VIROME_S4_L001_R2.fastq	FASTQ	DNA	4540936	556785316	50	122.6	151
S4-1876-viromeTruSeq.fastq	FASTQ	DNA	1639087	234162235	50	142.9	151
14-2820AC-VIROME_S5_L001_R1.fastq	FASTQ	DNA	3218914	379551536	50	117.9	151
14-2820AC-VIROME_S5_L001_R2.fastq	FASTQ	DNA	3218914	379232324	50	117.8	151
14-2820AC-viromeTruSeq.fastq	FASTQ	DNA	1333453	188490601	50	141.4	151
15-8466AC-VIROME_S6_L001_R1.fastq	FASTQ	DNA	3541076	443647313	50	125.3	151
15-8466AC-VIROME_S6_L001_R2.fastq	FASTQ	DNA	3541076	443317296	50	125.2	151
15-8466AC-viromeTruSeq.fastq	FASTQ	DNA	1321047	192023188	50	145.4	151
P1-2635-I-virome_R1.fastq	FASTQ	DNA	739396	83607983	50	113.1	151
P1-2635-I-virome_R2.fastq	FASTQ	DNA	739396	83400085	50	112.8	151

P1-2635-Q-virome_R1.fastq	FASTQ	DNA	856378	90701946	50	105.9	151
P1-2635-Q-virome_R2.fastq	FASTQ	DNA	856378	90521025	50	105.7	151
P1-2635-Ids-virome_R1.fastq	FASTQ	DNA	489337	55140137	50	112.7	151
P1-2635-Ids-virome_R2.fastq	FASTQ	DNA	489337	54935787	50	112.3	151
P2-6080-I-virome_R1.fastq	FASTQ	DNA	834110	100477574	50	120.5	151
P2-6080-I-virome_R2.fastq	FASTQ	DNA	834110	100402084	50	120.4	151
P2-6080-Q-virome_R1.fastq	FASTQ	DNA	904308	103042428	50	113.9	151
P2-6080-Q-virome_R2.fastq	FASTQ	DNA	904308	102909497	50	113.8	151
P2-6080-Ids-virome_R1.fastq	FASTQ	DNA	744404	90430974	50	121.5	151
P2-6080-Ids-virome_R2.fastq	FASTQ	DNA	744404	90359527	50	121.4	151
P3-5566-I-virome_R1.fastq	FASTQ	DNA	791244	95411484	50	120.6	151
P3-5566-I-virome_R2.fastq	FASTQ	DNA	791244	95306897	50	120.5	151
P3-5566-Q-virome_R1.fastq	FASTQ	DNA	804130	94598451	50	117.6	151
P3-5566-Q-virome_R2.fastq	FASTQ	DNA	804130	94476132	50	117.5	151
P3-5566-Ids-virome_R1.fastq	FASTQ	DNA	916744	111275067	50	121.4	151
P3-5566-Ids-virome_R2.fastq	FASTQ	DNA	916744	111184608	50	121.3	151
P4-1728-I-virome_R1.fastq	FASTQ	DNA	908923	108693950	50	119.6	151
P4-1728-I-virome_R2.fastq	FASTQ	DNA	908923	108614694	50	119.5	151
P4-1728-Q-virome_R1.fastq	FASTQ	DNA	880027	99717849	50	113.3	151
P4-1728-Q-virome_R2.fastq	FASTQ	DNA	880027	99584378	50	113.2	151
P4-1728-Ids-virome_R1.fastq	FASTQ	DNA	906062	109312118	50	120.6	151
P4-1728-Ids-virome_R2.fastq	FASTQ	DNA	906062	109226587	50	120.6	151
P5-8986-I-virome_R1.fastq	FASTQ	DNA	693210	83924257	50	121.1	151
P5-8986-I-virome_R2.fastq	FASTQ	DNA	693210	83841291	50	120.9	151
P5-8986-Q-virome_R1.fastq	FASTQ	DNA	821949	101157525	50	123.1	151
P5-8986-Q-virome_R2.fastq	FASTQ	DNA	821949	101073179	50	123	151
P5-8986-Ids-virome_R1.fastq	FASTQ	DNA	815061	97085018	50	119.1	151
P5-8986-Ids-virome_R2.fastq	FASTQ	DNA	815061	96956914	50	119	151
P6-8992-I-virome_R1.fastq	FASTQ	DNA	789156	94368196	50	119.6	151
P6-8992-I-virome_R2.fastq	FASTQ	DNA	789156	94291411	50	119.5	151

P6-8992-Q-virome_R1.fastq	FASTQ	DNA	681266	82094810	50	120.5	151
P6-8992-Q-virome_R2.fastq	FASTQ	DNA	681266	82024261	50	120.4	151
P6-8992-Ids-virome_R1.fastq	FASTQ	DNA	987145	117667768	50	119.2	151
P6-8992-Ids-virome_R2.fastq	FASTQ	DNA	987145	117572718	50	119.1	151

file	format	type	num_seqs	sum_len	min_len	avg_len	max_len
9092-U-virome_S11_L001_R1.fastq	FASTQ	DNA	1416217	150818638	50	106.5	151
9092-U-virome_S11_L001_R2.fastq	FASTQ	DNA	1416217	150518841	50	106.3	151
9092-1Q-virome_S3_L001_R1.fastq	FASTQ	DNA	1416111	134805045	50	95.2	151
9092-1Q-virome_S3_L001_R2.fastq	FASTQ	DNA	1416111	134440192	50	94.9	151
9092-2Z-virome_S1_L001_R1.fastq	FASTQ	DNA	1181208	117230893	50	99.2	151
9092-2Z-virome_S1_L001_R2.fastq	FASTQ	DNA	1181208	116974739	50	99	151
2820-U-virome_S14_L001_R1.fastq	FASTQ	DNA	1445127	158420790	50	109.6	151
2820-U-virome_S14_L001_R2.fastq	FASTQ	DNA	1445127	158149002	50	109.4	151
2820-2Q-virome_S8_L001_R1.fastq	FASTQ	DNA	1261752	124271931	50	98.5	151
2820-2Q-virome_S8_L001_R2.fastq	FASTQ	DNA	1261752	123938317	50	98.2	151
2820-2Z-virome_S2_L001_R1.fastq	FASTQ	DNA	1510189	165376165	50	109.5	151
2820-2Z-virome_S2_L001_R2.fastq	FASTQ	DNA	1510189	165153886	50	109.4	151
9324-U-virome_S12_L001_R1.fastq	FASTQ	DNA	1195768	120498045	50	100.8	151
9324-U-virome_S12_L001_R2.fastq	FASTQ	DNA	1195768	120136378	50	100.5	151
9324-2Q-virome_S5_L001_R1.fastq	FASTQ	DNA	1396180	155492855	50	111.4	151
9324-2Q-virome_S5_L001_R2.fastq	FASTQ	DNA	1396180	155188750	50	111.2	151
9324-2Z-virome_S4_L001_R1.fastq	FASTQ	DNA	1190404	127267155	50	106.9	151
9324-2Z-virome_S4_L001_R2.fastq	FASTQ	DNA	1190404	126952209	50	106.6	151
0168-U-virome_S13_L001_R1.fastq	FASTQ	DNA	1326604	145438214	50	109.6	151
0168-U-virome_S13_L001_R2.fastq	FASTQ	DNA	1326604	145153535	50	109.4	151
0168-1Q-virome_S7_L001_R1.fastq	FASTQ	DNA	1296291	140361276	50	108.3	151
0168-1Q-virome_S7_L001_R2.fastq	FASTQ	DNA	1296291	140054325	50	108	151
0168-1Z-virome_S6_L001_R1.fastq	FASTQ	DNA	1588890	163567631	50	102.9	151
0168-1Z-virome_S6_L001_R2.fastq	FASTQ	DNA	1588890	163236313	50	102.7	151
8466-U-virome_S17_L001_R1.fastq	FASTQ	DNA	1356243	152190019	50	112.2	151
8466-U-virome_S17_L001_R2.fastq	FASTQ	DNA	1356243	151966594	50	112	151
8466-1Q-virome_S10_L001_R1.fastq	FASTQ	DNA	1366812	132788288	50	97.2	151
8466-1Q-virome_S10_L001_R2.fastq	FASTQ	DNA	1366812	132414153	50	96.9	151
8466-2Z-virome_S9_L001_R1.fastq	FASTQ	DNA	1236579	131197456	50	106.1	151

8466-2Z-virome_S9_L001_R2.fastq	FASTQ	DNA	1236579	130893437	50	105.9	151
SAC1-viromeR_S55_L001_R1.fastq	FASTQ	DNA	331713	39030840	50	117.7	151
SAC1-viromeR_S55_L001_R2.fastq	FASTQ	DNA	331713	38973890	50	117.5	151
SAC2-viromeR_S56_L001_R1.fastq	FASTQ	DNA	337074	40041317	50	118.8	151
SAC2-viromeR_S56_L001_R2.fastq	FASTQ	DNA	337074	39983839	50	118.6	151
SBC1-virome_R1.fastq	FASTQ	DNA	283111	33723241	50	119.1	151
SBC1-virome_R2.fastq	FASTQ	DNA	283111	33679378	50	119	151
SBC2-virome_R1.fastq	FASTQ	DNA	293152	34505773	50	117.7	151
SBC2-virome_R2.fastq	FASTQ	DNA	293152	34460656	50	117.6	151
SAD1-viromeR_S61_L001_R1.fastq	FASTQ	DNA	213847	27268918	50	127.5	151
SAD1-viromeR_S61_L001_R2.fastq	FASTQ	DNA	213847	27235818	50	127.4	151
SAD2-virome_R1.fastq	FASTQ	DNA	244472	31469024	50	128.7	151
SAD2-virome_R2.fastq	FASTQ	DNA	244472	31438830	50	128.6	151
S1-5393-VIROME_S1_L001_R1.fastq	FASTQ	DNA	4614332	539714771	50	117	151
S1-5393-VIROME_S1_L001_R2.fastq	FASTQ	DNA	4614332	538933963	50	116.8	151
S1-5393-viromeTruSeq.fastq	FASTQ	DNA	1623594	237215689	50	146.1	151
S3-9504-VIROME_S3_L001_R1.fastq	FASTQ	DNA	4431859	539433655	50	121.7	151
S3-9504-VIROME_S3_L001_R2.fastq	FASTQ	DNA	4431859	538880314	50	121.6	151
S3-9504-viromeTruSeq.fastq	FASTQ	DNA	1020885	132461922	50	129.8	151
S4-1876-VIROME_S4_L001_R1.fastq	FASTQ	DNA	4540620	557276227	50	122.7	151
S4-1876-VIROME_S4_L001_R2.fastq	FASTQ	DNA	4540620	556764782	50	122.6	151
S4-1876-viromeTruSeq.fastq	FASTQ	DNA	1638975	234150063	50	142.9	151
14-2820AC-VIROME_S5_L001_R1.fas	FASTQ	DNA	3215899	379364407	50	118	151
14-2820AC-VIROME_S5_L001_R2.fas	FASTQ	DNA	3215899	379045258	50	117.9	151
14-2820AC-viromeTruSeq.fastq	FASTQ	DNA	1333118	188451951	50	141.4	151
15-8466AC-VIROME_S6_L001_R1.fas	FASTQ	DNA	3540653	443602853	50	125.3	151
15-8466AC-VIROME_S6_L001_R2.fas	FASTQ	DNA	3540653	443272736	50	125.2	151
15-8466AC-viromeTruSeq.fastq	FASTQ	DNA	1320934	192010897	50	145.4	151
P1-2635-I-virome_R1.fastq	FASTQ	DNA	699408	80714433	50	115.4	151
P1-2635-I-virome_R2.fastq	FASTQ	DNA	699408	80524211	50	115.1	151

P1-2635-Q-virome_R1.fastq	FASTQ	DNA	852429	90432867	50	106.1	151
P1-2635-Q-virome_R2.fastq	FASTQ	DNA	852429	90253161	50	105.9	151
P1-2635-Ids-virome_R1.fastq	FASTQ	DNA	441083	51649165	50	117.1	151
P1-2635-Ids-virome_R2.fastq	FASTQ	DNA	441083	51471458	50	116.7	151
P2-6080-I-virome_R1.fastq	FASTQ	DNA	832061	100333759	50	120.6	151
P2-6080-I-virome_R2.fastq	FASTQ	DNA	832061	100258642	50	120.5	151
P2-6080-Q-virome_R1.fastq	FASTQ	DNA	903894	103014545	50	114	151
P2-6080-Q-virome_R2.fastq	FASTQ	DNA	903894	102881755	50	113.8	151
P2-6080-Ids-virome_R1.fastq	FASTQ	DNA	740278	90141001	50	121.8	151
P2-6080-Ids-virome_R2.fastq	FASTQ	DNA	740278	90071428	50	121.7	151
P3-5566-I-virome_R1.fastq	FASTQ	DNA	790320	95346188	50	120.6	151
P3-5566-I-virome_R2.fastq	FASTQ	DNA	790320	95242136	50	120.5	151
P3-5566-Q-virome_R1.fastq	FASTQ	DNA	803360	94546066	50	117.7	151
P3-5566-Q-virome_R2.fastq	FASTQ	DNA	803360	94424009	50	117.5	151
P3-5566-Ids-virome_R1.fastq	FASTQ	DNA	914573	111123518	50	121.5	151
P3-5566-Ids-virome_R2.fastq	FASTQ	DNA	914573	111033843	50	121.4	151
P4-1728-I-virome_R1.fastq	FASTQ	DNA	908137	108638426	50	119.6	151
P4-1728-I-virome_R2.fastq	FASTQ	DNA	908137	108559729	50	119.5	151
P4-1728-Q-virome_R1.fastq	FASTQ	DNA	879077	99651340	50	113.4	151
P4-1728-Q-virome_R2.fastq	FASTQ	DNA	879077	99518234	50	113.2	151
P4-1728-Ids-virome_R1.fastq	FASTQ	DNA	904677	109211754	50	120.7	151
P4-1728-Ids-virome_R2.fastq	FASTQ	DNA	904677	109126842	50	120.6	151
P5-8986-I-virome_R1.fastq	FASTQ	DNA	689542	83661825	50	121.3	151
P5-8986-I-virome_R2.fastq	FASTQ	DNA	689542	83580389	50	121.2	151
P5-8986-Q-virome_R1.fastq	FASTQ	DNA	819192	100960457	50	123.2	151
P5-8986-Q-virome_R2.fastq	FASTQ	DNA	819192	100878043	50	123.1	151
P5-8986-Ids-virome_R1.fastq	FASTQ	DNA	803465	96233288	50	119.8	151
P5-8986-Ids-virome_R2.fastq	FASTQ	DNA	803465	96115897	50	119.6	151
P6-8992-I-virome_R1.fastq	FASTQ	DNA	788369	94310985	50	119.6	151
P6-8992-I-virome_R2.fastq	FASTQ	DNA	788369	94234601	50	119.5	151

P6-8992-Q-virome_R1.fastq	FASTQ	DNA	680667	82053389	50	120.5	151
P6-8992-Q-virome_R2.fastq	FASTQ	DNA	680667	81983306	50	120.4	151
P6-8992-Ids-virome_R1.fastq	FASTQ	DNA	985224	117530364	50	119.3	151
P6-8992-Ids-virome_R2.fastq	FASTQ	DNA	985224	117436544	50	119.2	151

file	format	type	num_seqs	sum_len	min_len	avg_len	max_len
9092-U-virome_S11_L001_R1.fastq	FASTQ	DNA	1664460	163186954	35	98	151
9092-U-virome_S11_L001_R2.fastq	FASTQ	DNA	1664460	163495575	35	98.2	151
9092-1Q-virome_S3_L001_R1.fastq	FASTQ	DNA	1742277	151362771	35	86.9	151
9092-1Q-virome_S3_L001_R2.fastq	FASTQ	DNA	1742277	151947123	35	87.2	151
9092-2Z-virome_S1_L001_R1.fastq	FASTQ	DNA	1456005	131401478	35	90.2	151
9092-2Z-virome_S1_L001_R2.fastq	FASTQ	DNA	1456005	131680471	35	90.4	151
2820-U-virome_S14_L001_R1.fastq	FASTQ	DNA	1691399	171695369	35	101.5	151
2820-U-virome_S14_L001_R2.fastq	FASTQ	DNA	1691399	171895716	35	101.6	151
2820-2Q-virome_S8_L001_R1.fastq	FASTQ	DNA	1460188	134566658	35	92.2	151
2820-2Q-virome_S8_L001_R2.fastq	FASTQ	DNA	1460188	135030567	35	92.5	151
2820-2Z-virome_S2_L001_R1.fastq	FASTQ	DNA	1697036	174879989	35	103.1	151
2820-2Z-virome_S2_L001_R2.fastq	FASTQ	DNA	1697036	175193919	35	103.2	151
9324-U-virome_S12_L001_R1.fastq	FASTQ	DNA	1600505	148133815	35	92.6	151
9324-U-virome_S12_L001_R2.fastq	FASTQ	DNA	1600505	148572861	35	92.8	151
9324-2Q-virome_S5_L001_R1.fastq	FASTQ	DNA	1542093	163786013	35	106.2	151
9324-2Q-virome_S5_L001_R2.fastq	FASTQ	DNA	1542093	164411454	35	106.6	151
9324-2Z-virome_S4_L001_R1.fastq	FASTQ	DNA	1375155	137902445	35	100.3	151
9324-2Z-virome_S4_L001_R2.fastq	FASTQ	DNA	1375155	138305700	35	100.6	151
0168-U-virome_S13_L001_R1.fastq	FASTQ	DNA	1538372	157572702	35	102.4	151
0168-U-virome_S13_L001_R2.fastq	FASTQ	DNA	1538372	157771802	35	102.6	151
0168-1Q-virome_S7_L001_R1.fastq	FASTQ	DNA	1499517	151049673	35	100.7	151
0168-1Q-virome_S7_L001_R2.fastq	FASTQ	DNA	1499517	151305475	35	100.9	151
0168-1Z-virome_S6_L001_R1.fastq	FASTQ	DNA	1862747	177209501	35	95.1	151
0168-1Z-virome_S6_L001_R2.fastq	FASTQ	DNA	1862747	177469015	35	95.3	151
8466-U-virome_S17_L001_R1.fastq	FASTQ	DNA	1555191	163086310	35	104.9	151
8466-U-virome_S17_L001_R2.fastq	FASTQ	DNA	1555191	163322109	35	105	151
8466-1Q-virome_S10_L001_R1.fastq	FASTQ	DNA	1673012	147733332	35	88.3	151
8466-1Q-virome_S10_L001_R2.fastq	FASTQ	DNA	1673012	148159065	35	88.6	151
8466-2Z-virome_S9_L001_R1.fastq	FASTQ	DNA	1410253	140124332	35	99.4	151

8466-2Z-virome_S9_L001_R2.fastq	FASTQ	DNA	1410253	140360043	35	99.5	151
SAC1-viromeR_S55_L001_R1.fastq	FASTQ	DNA	365571	40885137	35	111.8	151
SAC1-viromeR_S55_L001_R2.fastq	FASTQ	DNA	365571	40986018	35	112.1	151
SAC2-viromeR_S56_L001_R1.fastq	FASTQ	DNA	368602	41778370	35	113.3	151
SAC2-viromeR_S56_L001_R2.fastq	FASTQ	DNA	368602	41867666	35	113.6	151
SBC1-virome_R1.fastq	FASTQ	DNA	307751	35187033	35	114.3	151
SBC1-virome_R2.fastq	FASTQ	DNA	307751	35264133	35	114.6	151
SBC2-virome_R1.fastq	FASTQ	DNA	321036	36081076	35	112.4	151
SBC2-virome_R2.fastq	FASTQ	DNA	321036	36164267	35	112.6	151
SAD1-viromeR_S61_L001_R1.fastq	FASTQ	DNA	227262	28121635	35	123.7	151
SAD1-viromeR_S61_L001_R2.fastq	FASTQ	DNA	227262	28171300	35	124	151
SAD2-virome_R1.fastq	FASTQ	DNA	260881	32543242	35	124.7	151
SAD2-virome_R2.fastq	FASTQ	DNA	260881	32585940	35	124.9	151
S1-5393-VIROME_S1_L001_R1.fastq	FASTQ	DNA	5131783	570255202	35	111.1	151
S1-5393-VIROME_S1_L001_R2.fastq	FASTQ	DNA	5131783	571449917	35	111.4	151
S1-5393-viromeTruSeq.fastq	FASTQ	DNA	1654933	242120533	35	146.3	151
S3-9504-VIROME_S3_L001_R1.fastq	FASTQ	DNA	4781559	561014926	35	117.3	151
S3-9504-VIROME_S3_L001_R2.fastq	FASTQ	DNA	4781559	561767293	35	117.5	151
S3-9504-viromeTruSeq.fastq	FASTQ	DNA	1217574	161163452	35	132.4	151
S4-1876-VIROME_S4_L001_R1.fastq	FASTQ	DNA	4913300	578219441	35	117.7	151
S4-1876-VIROME_S4_L001_R2.fastq	FASTQ	DNA	4913300	578866901	35	117.8	151
S4-1876-viromeTruSeq.fastq	FASTQ	DNA	1694898	242602985	35	143.1	151
14-2820AC-VIROME_S5_L001_R1.fastq	FASTQ	DNA	3608775	402953356	35	111.7	151
14-2820AC-VIROME_S5_L001_R2.fastq	FASTQ	DNA	3608775	403287252	35	111.8	151
14-2820AC-viromeTruSeq.fastq	FASTQ	DNA	1390669	197636203	35	142.1	151
15-8466AC-VIROME_S6_L001_R1.fastq	FASTQ	DNA	3811556	460210413	35	120.7	151
15-8466AC-VIROME_S6_L001_R2.fastq	FASTQ	DNA	3811556	460772911	35	120.9	151
15-8466AC-viromeTruSeq.fastq	FASTQ	DNA	1350120	197017780	35	145.9	151
P1-2635-I-virome_R1.fastq	FASTQ	DNA	897795	99878387	35	111.2	151
P1-2635-I-virome_R2.fastq	FASTQ	DNA	897795	100125848	35	111.5	151

P1-2635-Q-virome_R1.fastq	FASTQ	DNA	1020060	100205640	35	98.2	151
P1-2635-Q-virome_R2.fastq	FASTQ	DNA	1020060	100449102	35	98.5	151
P1-2635-Ids-virome_R1.fastq	FASTQ	DNA	651458	73893996	35	113.4	151
P1-2635-Ids-virome_R2.fastq	FASTQ	DNA	651458	74219429	35	113.9	151
P2-6080-I-virome_R1.fastq	FASTQ	DNA	919576	105645784	35	114.9	151
P2-6080-I-virome_R2.fastq	FASTQ	DNA	919576	105752314	35	115	151
P2-6080-Q-virome_R1.fastq	FASTQ	DNA	1018526	109113824	35	107.1	151
P2-6080-Q-virome_R2.fastq	FASTQ	DNA	1018526	109229682	35	107.2	151
P2-6080-Ids-virome_R1.fastq	FASTQ	DNA	830553	96425469	35	116.1	151
P2-6080-Ids-virome_R2.fastq	FASTQ	DNA	830553	96502866	35	116.2	151
P3-5566-I-virome_R1.fastq	FASTQ	DNA	869113	99885773	35	114.9	151
P3-5566-I-virome_R2.fastq	FASTQ	DNA	869113	99970075	35	115	151
P3-5566-Q-virome_R1.fastq	FASTQ	DNA	895461	99874844	35	111.5	151
P3-5566-Q-virome_R2.fastq	FASTQ	DNA	895461	99977651	35	111.6	151
P3-5566-Ids-virome_R1.fastq	FASTQ	DNA	1018301	117549835	35	115.4	151
P3-5566-Ids-virome_R2.fastq	FASTQ	DNA	1018301	117660650	35	115.5	151
P4-1728-I-virome_R1.fastq	FASTQ	DNA	999725	113844547	35	113.9	151
P4-1728-I-virome_R2.fastq	FASTQ	DNA	999725	113966250	35	114	151
P4-1728-Q-virome_R1.fastq	FASTQ	DNA	969928	104680445	35	107.9	151
P4-1728-Q-virome_R2.fastq	FASTQ	DNA	969928	104790573	35	108	151
P4-1728-Ids-virome_R1.fastq	FASTQ	DNA	983342	114042200	35	116	151
P4-1728-Ids-virome_R2.fastq	FASTQ	DNA	983342	114146622	35	116.1	151
P5-8986-I-virome_R1.fastq	FASTQ	DNA	758798	88563713	35	116.7	151
P5-8986-I-virome_R2.fastq	FASTQ	DNA	758798	88653215	35	116.8	151
P5-8986-Q-virome_R1.fastq	FASTQ	DNA	891602	106335392	35	119.3	151
P5-8986-Q-virome_R2.fastq	FASTQ	DNA	891602	106413799	35	119.4	151
P5-8986-Ids-virome_R1.fastq	FASTQ	DNA	903287	104366876	35	115.5	151
P5-8986-Ids-virome_R2.fastq	FASTQ	DNA	903287	104467635	35	115.7	151
P6-8992-I-virome_R1.fastq	FASTQ	DNA	873992	99076446	35	113.4	151
P6-8992-I-virome_R2.fastq	FASTQ	DNA	873992	99149221	35	113.4	151

P6-8992-Q-virome_R1.fastq	FASTQ	DNA	746119	86020732	35	115.3	151
P6-8992-Q-virome_R2.fastq	FASTQ	DNA	746119	86107475	35	115.4	151
P6-8992-Ids-virome_R1.fastq	FASTQ	DNA	1103322	124352570	35	112.7	151
P6-8992-Ids-virome_R2.fastq	FASTQ	DNA	1103322	124414225	35	112.8	151
