# Supplementary material for: A comparison of methods for the optimal recovery of the human fecal virome
Source: ISME Commun. 2026 Apr 11;6(1):ycag090. doi: 10.1093/ismeco/ycag090 (PMC13155102; doi:10.1093/ismeco/ycag090)
Supplement: Suppl_material_updated_ycag090 [file suppl_material_updated_ycag090.pdf]

		Fastp	Human Read Removal	
**file**	**Raw Reads (R1)**	**Reads After**	**Reads Removed**	**Reads After**
9092-U-virome_S11_L001_R1.fastq	1664460	1417212	995	1416217
9092-1Q-virome_S3_L001_R1.fastq	1742277	1419592	3481	1416111
9092-2Z-virome_S1_L001_R1.fastq	1456005	1181383	175	1181208
2820-U-virome_S14_L001_R1.fastq	1691399	1447463	2336	1445127
2820-2Q-virome_S8_L001_R1.fastq	1460188	1261848	96	1261752
2820-2Z-virome_S2_L001_R1.fastq	1697036	1510265	76	1510189
9324-U-virome_S12_L001_R1.fastq	1600505	1213434	17666	1195768
9324-2Q-virome_S5_L001_R1.fastq	1542093	1396271	91	1396180
9324-2Z-virome_S4_L001_R1.fastq	1375155	1191969	1565	1190404
0168-U-virome_S13_L001_R1.fastq	1538372	1329715	3111	1326604
0168-1Q-virome_S7_L001_R1.fastq	1499517	1297634	1343	1296291
0168-1Z-virome_S6_L001_R1.fastq	1862747	1591820	2930	1588890
8466-U-virome_S17_L001_R1.fastq	1555191	1356565	322	1356243
8466-1Q-virome_S10_L001_R1.fastq	1673012	1367067	255	1366812
8466-2Z-virome_S9_L001_R1.fastq	1410253	1237346	767	1236579
SAC1-viromeR_S55_L001_R1.fastq	365571	331725	12	331713
SAC2-viromeR_S56_L001_R1.fastq	368602	337085	11	337074
SBC1-virome_R1.fastq	307751	283223	112	283111
SBC2-virome_R1.fastq	321036	293170	18	293152
SAD1-viromeR_S61_L001_R1.fastq	227262	213851	4	213847
SAD2-virome_R1.fastq	260881	244483	11	244472
S1-5393-VIROME_S1_L001_R1.fastq	5131783	4617437	3105	4614332
S1-5393-viromeTruSeq.fastq	1654933	1624596	1002	1623594
S3-9504-VIROME_S3_L001_R1.fastq	4781559	4434669	2810	4431859
S3-9504-viromeTruSeq.fastq	1217574	1021570	685	1020885
S4-1876-VIROME_S4_L001_R1.fastq	4913300	4540936	316	4540620
S4-1876-viromeTruSeq.fastq	1694898	1639087	112	1638975
14-2820AC-VIROME_S5_L001_R1.fastq	3608775	3218914	3015	3215899

14-2820AC-viromeTruSeq.fastq	1390669	1333453	335	1333118
15-8466AC-VIROME_S6_L001_R1.fastq	3811556	3541076	423	3540653
15-8466AC-viromeTruSeq.fastq	1350120	1321047	113	1320934
P1-2635-I-virome_R1.fastq	897795	739396	39988	699408
P1-2635-Q-virome_R1.fastq	1020060	856378	3949	852429
P1-2635-Ids-virome_R1.fastq	651458	489337	48254	441083
P2-6080-I-virome_R1.fastq	919576	834110	2049	832061
P2-6080-Q-virome_R1.fastq	1018526	904308	414	903894
P2-6080-Ids-virome_R1.fastq	830553	744404	4126	740278
P3-5566-I-virome_R1.fastq	869113	791244	924	790320
P3-5566-Q-virome_R1.fastq	895461	804130	770	803360
P3-5566-Ids-virome_R1.fastq	1018301	916744	2171	914573
P4-1728-I-virome_R1.fastq	999725	908923	786	908137
P4-1728-Q-virome_R1.fastq	969928	880027	950	879077
P4-1728-Ids-virome_R1.fastq	983342	906062	1385	904677
P5-8986-I-virome_R1.fastq	758798	693210	3668	689542
P5-8986-Q-virome_R1.fastq	891602	821949	2757	819192
P5-8986-Ids-virome_R1.fastq	903287	815061	11596	803465
P6-8992-I-virome_R1.fastq	873992	789156	787	788369
P6-8992-Q-virome_R1.fastq	746119	681266	599	680667
P6-8992-Ids-virome_R1.fastq	1103322	987145	1921	985224

file	format	type	Reads After Fastp	sum_len	min_len	avg_len	max_len
9092-U-virome_S11_L001_R1.fastq	FASTQ	DNA	1417212	150912234	50	106.5	151
9092-U-virome_S11_L001_R2.fastq	FASTQ	DNA	1417212	150612398	50	106.3	151
9092-1Q-virome_S3_L001_R1.fastq	FASTQ	DNA	1419592	135065033	50	95.1	151
9092-1Q-virome_S3_L001_R2.fastq	FASTQ	DNA	1419592	134676676	50	94.9	151
9092-2Z-virome_S1_L001_R1.fastq	FASTQ	DNA	1181383	117247142	50	99.2	151
9092-2Z-virome_S1_L001_R2.fastq	FASTQ	DNA	1181383	116990968	50	99	151
2820-U-virome_S14_L001_R1.fastq	FASTQ	DNA	1447463	158570239	50	109.6	151
2820-U-virome_S14_L001_R2.fastq	FASTQ	DNA	1447463	158298344	50	109.4	151
2820-2Q-virome_S8_L001_R1.fastq	FASTQ	DNA	1261848	124280606	50	98.5	151
2820-2Q-virome_S8_L001_R2.fastq	FASTQ	DNA	1261848	123946963	50	98.2	151
2820-2Z-virome_S2_L001_R1.fastq	FASTQ	DNA	1510265	165383692	50	109.5	151
2820-2Z-virome_S2_L001_R2.fastq	FASTQ	DNA	1510265	165161416	50	109.4	151
9324-U-virome_S12_L001_R1.fastq	FASTQ	DNA	1213434	121680025	50	100.3	151
9324-U-virome_S12_L001_R2.fastq	FASTQ	DNA	1213434	121315226	50	100	151
9324-2Q-virome_S5_L001_R1.fastq	FASTQ	DNA	1396271	155501595	50	111.4	151
9324-2Q-virome_S5_L001_R2.fastq	FASTQ	DNA	1396271	155197498	50	111.2	151
9324-2Z-virome_S4_L001_R1.fastq	FASTQ	DNA	1191969	127427880	50	106.9	151
9324-2Z-virome_S4_L001_R2.fastq	FASTQ	DNA	1191969	127112831	50	106.6	151
0168-U-virome_S13_L001_R1.fastq	FASTQ	DNA	1329715	145641658	50	109.5	151
0168-U-virome_S13_L001_R2.fastq	FASTQ	DNA	1329715	145356512	50	109.3	151
0168-1Q-virome_S7_L001_R1.fastq	FASTQ	DNA	1297634	140486614	50	108.3	151
0168-1Q-virome_S7_L001_R2.fastq	FASTQ	DNA	1297634	140179597	50	108	151
0168-1Z-virome_S6_L001_R1.fastq	FASTQ	DNA	1591820	163870803	50	102.9	151
0168-1Z-virome_S6_L001_R2.fastq	FASTQ	DNA	1591820	163539449	50	102.7	151
8466-U-virome_S17_L001_R1.fastq	FASTQ	DNA	1356565	152210113	50	112.2	151
8466-U-virome_S17_L001_R2.fastq	FASTQ	DNA	1356565	151986613	50	112	151
8466-1Q-virome_S10_L001_R1.fastq	FASTQ	DNA	1367067	132809156	50	97.1	151
8466-1Q-virome_S10_L001_R2.fastq	FASTQ	DNA	1367067	132435012	50	96.9	151
8466-2Z-virome_S9_L001_R1.fastq	FASTQ	DNA	1237346	131272792	50	106.1	151

8466-2Z-virome_S9_L001_R2.fastq	FASTQ	DNA	1237346	130968555	50	105.8	151
SAC1-viromeR_S55_L001_R1.fastq	FASTQ	DNA	331725	39031581	50	117.7	151
SAC1-viromeR_S55_L001_R2.fastq	FASTQ	DNA	331725	38974631	50	117.5	151
SAC2-viromeR_S56_L001_R1.fastq	FASTQ	DNA	337085	40042025	50	118.8	151
SAC2-viromeR_S56_L001_R2.fastq	FASTQ	DNA	337085	39984546	50	118.6	151
SBC1-virome_R1.fastq	FASTQ	DNA	283223	33736104	50	119.1	151
SBC1-virome_R2.fastq	FASTQ	DNA	283223	33692234	50	119	151
SBC2-virome_R1.fastq	FASTQ	DNA	293170	34506971	50	117.7	151
SBC2-virome_R2.fastq	FASTQ	DNA	293170	34461852	50	117.5	151
SAD1-viromeR_S61_L001_R1.fastq	FASTQ	DNA	213851	27269445	50	127.5	151
SAD1-viromeR_S61_L001_R2.fastq	FASTQ	DNA	213851	27236344	50	127.4	151
SAD2-virome_R1.fastq	FASTQ	DNA	244483	31470425	50	128.7	151
SAD2-virome_R2.fastq	FASTQ	DNA	244483	31440229	50	128.6	151
S1-5393-VIROME_S1_L001_R1.fastq	FASTQ	DNA	4617437	539934391	50	116.9	151
S1-5393-VIROME_S1_L001_R2.fastq	FASTQ	DNA	4617437	539152616	50	116.8	151
S1-5393-viromeTruSeq.fastq	FASTQ	DNA	1624596	237353678	50	146.1	151
S3-9504-VIROME_S3_L001_R1.fastq	FASTQ	DNA	4434669	539628515	50	121.7	151
S3-9504-VIROME_S3_L001_R2.fastq	FASTQ	DNA	4434669	539073611	50	121.6	151
S3-9504-viromeTruSeq.fastq	FASTQ	DNA	1021570	132551922	50	129.8	151
S4-1876-VIROME_S4_L001_R1.fastq	FASTQ	DNA	4540936	557296874	50	122.7	151
S4-1876-VIROME_S4_L001_R2.fastq	FASTQ	DNA	4540936	556785316	50	122.6	151
S4-1876-viromeTruSeq.fastq	FASTQ	DNA	1639087	234162235	50	142.9	151
14-2820AC-VIROME_S5_L001_R1.fastq	FASTQ	DNA	3218914	379551536	50	117.9	151
14-2820AC-VIROME_S5_L001_R2.fastq	FASTQ	DNA	3218914	379232324	50	117.8	151
14-2820AC-viromeTruSeq.fastq	FASTQ	DNA	1333453	188490601	50	141.4	151
15-8466AC-VIROME_S6_L001_R1.fastq	FASTQ	DNA	3541076	443647313	50	125.3	151
15-8466AC-VIROME_S6_L001_R2.fastq	FASTQ	DNA	3541076	443317296	50	125.2	151
15-8466AC-viromeTruSeq.fastq	FASTQ	DNA	1321047	192023188	50	145.4	151
P1-2635-I-virome_R1.fastq	FASTQ	DNA	739396	83607983	50	113.1	151
P1-2635-I-virome_R2.fastq	FASTQ	DNA	739396	83400085	50	112.8	151

P1-2635-Q-virome_R1.fastq	FASTQ	DNA	856378	90701946	50	105.9	151
P1-2635-Q-virome_R2.fastq	FASTQ	DNA	856378	90521025	50	105.7	151
P1-2635-Ids-virome_R1.fastq	FASTQ	DNA	489337	55140137	50	112.7	151
P1-2635-Ids-virome_R2.fastq	FASTQ	DNA	489337	54935787	50	112.3	151
P2-6080-I-virome_R1.fastq	FASTQ	DNA	834110	100477574	50	120.5	151
P2-6080-I-virome_R2.fastq	FASTQ	DNA	834110	100402084	50	120.4	151
P2-6080-Q-virome_R1.fastq	FASTQ	DNA	904308	103042428	50	113.9	151
P2-6080-Q-virome_R2.fastq	FASTQ	DNA	904308	102909497	50	113.8	151
P2-6080-Ids-virome_R1.fastq	FASTQ	DNA	744404	90430974	50	121.5	151
P2-6080-Ids-virome_R2.fastq	FASTQ	DNA	744404	90359527	50	121.4	151
P3-5566-I-virome_R1.fastq	FASTQ	DNA	791244	95411484	50	120.6	151
P3-5566-I-virome_R2.fastq	FASTQ	DNA	791244	95306897	50	120.5	151
P3-5566-Q-virome_R1.fastq	FASTQ	DNA	804130	94598451	50	117.6	151
P3-5566-Q-virome_R2.fastq	FASTQ	DNA	804130	94476132	50	117.5	151
P3-5566-Ids-virome_R1.fastq	FASTQ	DNA	916744	111275067	50	121.4	151
P3-5566-Ids-virome_R2.fastq	FASTQ	DNA	916744	111184608	50	121.3	151
P4-1728-I-virome_R1.fastq	FASTQ	DNA	908923	108693950	50	119.6	151
P4-1728-I-virome_R2.fastq	FASTQ	DNA	908923	108614694	50	119.5	151
P4-1728-Q-virome_R1.fastq	FASTQ	DNA	880027	99717849	50	113.3	151
P4-1728-Q-virome_R2.fastq	FASTQ	DNA	880027	99584378	50	113.2	151
P4-1728-Ids-virome_R1.fastq	FASTQ	DNA	906062	109312118	50	120.6	151
P4-1728-Ids-virome_R2.fastq	FASTQ	DNA	906062	109226587	50	120.6	151
P5-8986-I-virome_R1.fastq	FASTQ	DNA	693210	83924257	50	121.1	151
P5-8986-I-virome_R2.fastq	FASTQ	DNA	693210	83841291	50	120.9	151
P5-8986-Q-virome_R1.fastq	FASTQ	DNA	821949	101157525	50	123.1	151
P5-8986-Q-virome_R2.fastq	FASTQ	DNA	821949	101073179	50	123	151
P5-8986-Ids-virome_R1.fastq	FASTQ	DNA	815061	97085018	50	119.1	151
P5-8986-Ids-virome_R2.fastq	FASTQ	DNA	815061	96956914	50	119	151
P6-8992-I-virome_R1.fastq	FASTQ	DNA	789156	94368196	50	119.6	151
P6-8992-I-virome_R2.fastq	FASTQ	DNA	789156	94291411	50	119.5	151

P6-8992-Q-virome_R1.fastq	FASTQ	DNA	681266	82094810	50	120.5	151
P6-8992-Q-virome_R2.fastq	FASTQ	DNA	681266	82024261	50	120.4	151
P6-8992-Ids-virome_R1.fastq	FASTQ	DNA	987145	117667768	50	119.2	151
P6-8992-Ids-virome_R2.fastq	FASTQ	DNA	987145	117572718	50	119.1	151

file	format	type	num_seqs	sum_len	min_len	avg_len	max_len
9092-U-virome_S11_L001_R1.fastq	FASTQ	DNA	1416217	150818638	50	106.5	151
9092-U-virome_S11_L001_R2.fastq	FASTQ	DNA	1416217	150518841	50	106.3	151
9092-1Q-virome_S3_L001_R1.fastq	FASTQ	DNA	1416111	134805045	50	95.2	151
9092-1Q-virome_S3_L001_R2.fastq	FASTQ	DNA	1416111	134440192	50	94.9	151
9092-2Z-virome_S1_L001_R1.fastq	FASTQ	DNA	1181208	117230893	50	99.2	151
9092-2Z-virome_S1_L001_R2.fastq	FASTQ	DNA	1181208	116974739	50	99	151
2820-U-virome_S14_L001_R1.fastq	FASTQ	DNA	1445127	158420790	50	109.6	151
2820-U-virome_S14_L001_R2.fastq	FASTQ	DNA	1445127	158149002	50	109.4	151
2820-2Q-virome_S8_L001_R1.fastq	FASTQ	DNA	1261752	124271931	50	98.5	151
2820-2Q-virome_S8_L001_R2.fastq	FASTQ	DNA	1261752	123938317	50	98.2	151
2820-2Z-virome_S2_L001_R1.fastq	FASTQ	DNA	1510189	165376165	50	109.5	151
2820-2Z-virome_S2_L001_R2.fastq	FASTQ	DNA	1510189	165153886	50	109.4	151
9324-U-virome_S12_L001_R1.fastq	FASTQ	DNA	1195768	120498045	50	100.8	151
9324-U-virome_S12_L001_R2.fastq	FASTQ	DNA	1195768	120136378	50	100.5	151
9324-2Q-virome_S5_L001_R1.fastq	FASTQ	DNA	1396180	155492855	50	111.4	151
9324-2Q-virome_S5_L001_R2.fastq	FASTQ	DNA	1396180	155188750	50	111.2	151
9324-2Z-virome_S4_L001_R1.fastq	FASTQ	DNA	1190404	127267155	50	106.9	151
9324-2Z-virome_S4_L001_R2.fastq	FASTQ	DNA	1190404	126952209	50	106.6	151
0168-U-virome_S13_L001_R1.fastq	FASTQ	DNA	1326604	145438214	50	109.6	151
0168-U-virome_S13_L001_R2.fastq	FASTQ	DNA	1326604	145153535	50	109.4	151
0168-1Q-virome_S7_L001_R1.fastq	FASTQ	DNA	1296291	140361276	50	108.3	151
0168-1Q-virome_S7_L001_R2.fastq	FASTQ	DNA	1296291	140054325	50	108	151
0168-1Z-virome_S6_L001_R1.fastq	FASTQ	DNA	1588890	163567631	50	102.9	151
0168-1Z-virome_S6_L001_R2.fastq	FASTQ	DNA	1588890	163236313	50	102.7	151
8466-U-virome_S17_L001_R1.fastq	FASTQ	DNA	1356243	152190019	50	112.2	151
8466-U-virome_S17_L001_R2.fastq	FASTQ	DNA	1356243	151966594	50	112	151
8466-1Q-virome_S10_L001_R1.fastq	FASTQ	DNA	1366812	132788288	50	97.2	151
8466-1Q-virome_S10_L001_R2.fastq	FASTQ	DNA	1366812	132414153	50	96.9	151
8466-2Z-virome_S9_L001_R1.fastq	FASTQ	DNA	1236579	131197456	50	106.1	151

8466-2Z-virome_S9_L001_R2.fastq	FASTQ	DNA	1236579	130893437	50	105.9	151
SAC1-viromeR_S55_L001_R1.fastq	FASTQ	DNA	331713	39030840	50	117.7	151
SAC1-viromeR_S55_L001_R2.fastq	FASTQ	DNA	331713	38973890	50	117.5	151
SAC2-viromeR_S56_L001_R1.fastq	FASTQ	DNA	337074	40041317	50	118.8	151
SAC2-viromeR_S56_L001_R2.fastq	FASTQ	DNA	337074	39983839	50	118.6	151
SBC1-virome_R1.fastq	FASTQ	DNA	283111	33723241	50	119.1	151
SBC1-virome_R2.fastq	FASTQ	DNA	283111	33679378	50	119	151
SBC2-virome_R1.fastq	FASTQ	DNA	293152	34505773	50	117.7	151
SBC2-virome_R2.fastq	FASTQ	DNA	293152	34460656	50	117.6	151
SAD1-viromeR_S61_L001_R1.fastq	FASTQ	DNA	213847	27268918	50	127.5	151
SAD1-viromeR_S61_L001_R2.fastq	FASTQ	DNA	213847	27235818	50	127.4	151
SAD2-virome_R1.fastq	FASTQ	DNA	244472	31469024	50	128.7	151
SAD2-virome_R2.fastq	FASTQ	DNA	244472	31438830	50	128.6	151
S1-5393-VIROME_S1_L001_R1.fastq	FASTQ	DNA	4614332	539714771	50	117	151
S1-5393-VIROME_S1_L001_R2.fastq	FASTQ	DNA	4614332	538933963	50	116.8	151
S1-5393-viromeTruSeq.fastq	FASTQ	DNA	1623594	237215689	50	146.1	151
S3-9504-VIROME_S3_L001_R1.fastq	FASTQ	DNA	4431859	539433655	50	121.7	151
S3-9504-VIROME_S3_L001_R2.fastq	FASTQ	DNA	4431859	538880314	50	121.6	151
S3-9504-viromeTruSeq.fastq	FASTQ	DNA	1020885	132461922	50	129.8	151
S4-1876-VIROME_S4_L001_R1.fastq	FASTQ	DNA	4540620	557276227	50	122.7	151
S4-1876-VIROME_S4_L001_R2.fastq	FASTQ	DNA	4540620	556764782	50	122.6	151
S4-1876-viromeTruSeq.fastq	FASTQ	DNA	1638975	234150063	50	142.9	151
14-2820AC-VIROME_S5_L001_R1.fas	FASTQ	DNA	3215899	379364407	50	118	151
14-2820AC-VIROME_S5_L001_R2.fas	FASTQ	DNA	3215899	379045258	50	117.9	151
14-2820AC-viromeTruSeq.fastq	FASTQ	DNA	1333118	188451951	50	141.4	151
15-8466AC-VIROME_S6_L001_R1.fas	FASTQ	DNA	3540653	443602853	50	125.3	151
15-8466AC-VIROME_S6_L001_R2.fas	FASTQ	DNA	3540653	443272736	50	125.2	151
15-8466AC-viromeTruSeq.fastq	FASTQ	DNA	1320934	192010897	50	145.4	151
P1-2635-I-virome_R1.fastq	FASTQ	DNA	699408	80714433	50	115.4	151
P1-2635-I-virome_R2.fastq	FASTQ	DNA	699408	80524211	50	115.1	151

P1-2635-Q-virome_R1.fastq	FASTQ	DNA	852429	90432867	50	106.1	151
P1-2635-Q-virome_R2.fastq	FASTQ	DNA	852429	90253161	50	105.9	151
P1-2635-Ids-virome_R1.fastq	FASTQ	DNA	441083	51649165	50	117.1	151
P1-2635-Ids-virome_R2.fastq	FASTQ	DNA	441083	51471458	50	116.7	151
P2-6080-I-virome_R1.fastq	FASTQ	DNA	832061	100333759	50	120.6	151
P2-6080-I-virome_R2.fastq	FASTQ	DNA	832061	100258642	50	120.5	151
P2-6080-Q-virome_R1.fastq	FASTQ	DNA	903894	103014545	50	114	151
P2-6080-Q-virome_R2.fastq	FASTQ	DNA	903894	102881755	50	113.8	151
P2-6080-Ids-virome_R1.fastq	FASTQ	DNA	740278	90141001	50	121.8	151
P2-6080-Ids-virome_R2.fastq	FASTQ	DNA	740278	90071428	50	121.7	151
P3-5566-I-virome_R1.fastq	FASTQ	DNA	790320	95346188	50	120.6	151
P3-5566-I-virome_R2.fastq	FASTQ	DNA	790320	95242136	50	120.5	151
P3-5566-Q-virome_R1.fastq	FASTQ	DNA	803360	94546066	50	117.7	151
P3-5566-Q-virome_R2.fastq	FASTQ	DNA	803360	94424009	50	117.5	151
P3-5566-Ids-virome_R1.fastq	FASTQ	DNA	914573	111123518	50	121.5	151
P3-5566-Ids-virome_R2.fastq	FASTQ	DNA	914573	111033843	50	121.4	151
P4-1728-I-virome_R1.fastq	FASTQ	DNA	908137	108638426	50	119.6	151
P4-1728-I-virome_R2.fastq	FASTQ	DNA	908137	108559729	50	119.5	151
P4-1728-Q-virome_R1.fastq	FASTQ	DNA	879077	99651340	50	113.4	151
P4-1728-Q-virome_R2.fastq	FASTQ	DNA	879077	99518234	50	113.2	151
P4-1728-Ids-virome_R1.fastq	FASTQ	DNA	904677	109211754	50	120.7	151
P4-1728-Ids-virome_R2.fastq	FASTQ	DNA	904677	109126842	50	120.6	151
P5-8986-I-virome_R1.fastq	FASTQ	DNA	689542	83661825	50	121.3	151
P5-8986-I-virome_R2.fastq	FASTQ	DNA	689542	83580389	50	121.2	151
P5-8986-Q-virome_R1.fastq	FASTQ	DNA	819192	100960457	50	123.2	151
P5-8986-Q-virome_R2.fastq	FASTQ	DNA	819192	100878043	50	123.1	151
P5-8986-Ids-virome_R1.fastq	FASTQ	DNA	803465	96233288	50	119.8	151
P5-8986-Ids-virome_R2.fastq	FASTQ	DNA	803465	96115897	50	119.6	151
P6-8992-I-virome_R1.fastq	FASTQ	DNA	788369	94310985	50	119.6	151
P6-8992-I-virome_R2.fastq	FASTQ	DNA	788369	94234601	50	119.5	151

P6-8992-Q-virome_R1.fastq	FASTQ	DNA	680667	82053389	50	120.5	151
P6-8992-Q-virome_R2.fastq	FASTQ	DNA	680667	81983306	50	120.4	151
P6-8992-Ids-virome_R1.fastq	FASTQ	DNA	985224	117530364	50	119.3	151
P6-8992-Ids-virome_R2.fastq	FASTQ	DNA	985224	117436544	50	119.2	151

file	format	type	num_seqs	sum_len	min_len	avg_len	max_len
9092-U-virome_S11_L001_R1.fastq	FASTQ	DNA	1664460	163186954	35	98	151
9092-U-virome_S11_L001_R2.fastq	FASTQ	DNA	1664460	163495575	35	98.2	151
9092-1Q-virome_S3_L001_R1.fastq	FASTQ	DNA	1742277	151362771	35	86.9	151
9092-1Q-virome_S3_L001_R2.fastq	FASTQ	DNA	1742277	151947123	35	87.2	151
9092-2Z-virome_S1_L001_R1.fastq	FASTQ	DNA	1456005	131401478	35	90.2	151
9092-2Z-virome_S1_L001_R2.fastq	FASTQ	DNA	1456005	131680471	35	90.4	151
2820-U-virome_S14_L001_R1.fastq	FASTQ	DNA	1691399	171695369	35	101.5	151
2820-U-virome_S14_L001_R2.fastq	FASTQ	DNA	1691399	171895716	35	101.6	151
2820-2Q-virome_S8_L001_R1.fastq	FASTQ	DNA	1460188	134566658	35	92.2	151
2820-2Q-virome_S8_L001_R2.fastq	FASTQ	DNA	1460188	135030567	35	92.5	151
2820-2Z-virome_S2_L001_R1.fastq	FASTQ	DNA	1697036	174879989	35	103.1	151
2820-2Z-virome_S2_L001_R2.fastq	FASTQ	DNA	1697036	175193919	35	103.2	151
9324-U-virome_S12_L001_R1.fastq	FASTQ	DNA	1600505	148133815	35	92.6	151
9324-U-virome_S12_L001_R2.fastq	FASTQ	DNA	1600505	148572861	35	92.8	151
9324-2Q-virome_S5_L001_R1.fastq	FASTQ	DNA	1542093	163786013	35	106.2	151
9324-2Q-virome_S5_L001_R2.fastq	FASTQ	DNA	1542093	164411454	35	106.6	151
9324-2Z-virome_S4_L001_R1.fastq	FASTQ	DNA	1375155	137902445	35	100.3	151
9324-2Z-virome_S4_L001_R2.fastq	FASTQ	DNA	1375155	138305700	35	100.6	151
0168-U-virome_S13_L001_R1.fastq	FASTQ	DNA	1538372	157572702	35	102.4	151
0168-U-virome_S13_L001_R2.fastq	FASTQ	DNA	1538372	157771802	35	102.6	151
0168-1Q-virome_S7_L001_R1.fastq	FASTQ	DNA	1499517	151049673	35	100.7	151
0168-1Q-virome_S7_L001_R2.fastq	FASTQ	DNA	1499517	151305475	35	100.9	151
0168-1Z-virome_S6_L001_R1.fastq	FASTQ	DNA	1862747	177209501	35	95.1	151
0168-1Z-virome_S6_L001_R2.fastq	FASTQ	DNA	1862747	177469015	35	95.3	151
8466-U-virome_S17_L001_R1.fastq	FASTQ	DNA	1555191	163086310	35	104.9	151
8466-U-virome_S17_L001_R2.fastq	FASTQ	DNA	1555191	163322109	35	105	151
8466-1Q-virome_S10_L001_R1.fastq	FASTQ	DNA	1673012	147733332	35	88.3	151
8466-1Q-virome_S10_L001_R2.fastq	FASTQ	DNA	1673012	148159065	35	88.6	151
8466-2Z-virome_S9_L001_R1.fastq	FASTQ	DNA	1410253	140124332	35	99.4	151

8466-2Z-virome_S9_L001_R2.fastq	FASTQ	DNA	1410253	140360043	35	99.5	151
SAC1-viromeR_S55_L001_R1.fastq	FASTQ	DNA	365571	40885137	35	111.8	151
SAC1-viromeR_S55_L001_R2.fastq	FASTQ	DNA	365571	40986018	35	112.1	151
SAC2-viromeR_S56_L001_R1.fastq	FASTQ	DNA	368602	41778370	35	113.3	151
SAC2-viromeR_S56_L001_R2.fastq	FASTQ	DNA	368602	41867666	35	113.6	151
SBC1-virome_R1.fastq	FASTQ	DNA	307751	35187033	35	114.3	151
SBC1-virome_R2.fastq	FASTQ	DNA	307751	35264133	35	114.6	151
SBC2-virome_R1.fastq	FASTQ	DNA	321036	36081076	35	112.4	151
SBC2-virome_R2.fastq	FASTQ	DNA	321036	36164267	35	112.6	151
SAD1-viromeR_S61_L001_R1.fastq	FASTQ	DNA	227262	28121635	35	123.7	151
SAD1-viromeR_S61_L001_R2.fastq	FASTQ	DNA	227262	28171300	35	124	151
SAD2-virome_R1.fastq	FASTQ	DNA	260881	32543242	35	124.7	151
SAD2-virome_R2.fastq	FASTQ	DNA	260881	32585940	35	124.9	151
S1-5393-VIROME_S1_L001_R1.fastq	FASTQ	DNA	5131783	570255202	35	111.1	151
S1-5393-VIROME_S1_L001_R2.fastq	FASTQ	DNA	5131783	571449917	35	111.4	151
S1-5393-viromeTruSeq.fastq	FASTQ	DNA	1654933	242120533	35	146.3	151
S3-9504-VIROME_S3_L001_R1.fastq	FASTQ	DNA	4781559	561014926	35	117.3	151
S3-9504-VIROME_S3_L001_R2.fastq	FASTQ	DNA	4781559	561767293	35	117.5	151
S3-9504-viromeTruSeq.fastq	FASTQ	DNA	1217574	161163452	35	132.4	151
S4-1876-VIROME_S4_L001_R1.fastq	FASTQ	DNA	4913300	578219441	35	117.7	151
S4-1876-VIROME_S4_L001_R2.fastq	FASTQ	DNA	4913300	578866901	35	117.8	151
S4-1876-viromeTruSeq.fastq	FASTQ	DNA	1694898	242602985	35	143.1	151
14-2820AC-VIROME_S5_L001_R1.fastq	FASTQ	DNA	3608775	402953356	35	111.7	151
14-2820AC-VIROME_S5_L001_R2.fastq	FASTQ	DNA	3608775	403287252	35	111.8	151
14-2820AC-viromeTruSeq.fastq	FASTQ	DNA	1390669	197636203	35	142.1	151
15-8466AC-VIROME_S6_L001_R1.fastq	FASTQ	DNA	3811556	460210413	35	120.7	151
15-8466AC-VIROME_S6_L001_R2.fastq	FASTQ	DNA	3811556	460772911	35	120.9	151
15-8466AC-viromeTruSeq.fastq	FASTQ	DNA	1350120	197017780	35	145.9	151
P1-2635-I-virome_R1.fastq	FASTQ	DNA	897795	99878387	35	111.2	151
P1-2635-I-virome_R2.fastq	FASTQ	DNA	897795	100125848	35	111.5	151

P1-2635-Q-virome_R1.fastq	FASTQ	DNA	1020060	100205640	35	98.2	151
P1-2635-Q-virome_R2.fastq	FASTQ	DNA	1020060	100449102	35	98.5	151
P1-2635-Ids-virome_R1.fastq	FASTQ	DNA	651458	73893996	35	113.4	151
P1-2635-Ids-virome_R2.fastq	FASTQ	DNA	651458	74219429	35	113.9	151
P2-6080-I-virome_R1.fastq	FASTQ	DNA	919576	105645784	35	114.9	151
P2-6080-I-virome_R2.fastq	FASTQ	DNA	919576	105752314	35	115	151
P2-6080-Q-virome_R1.fastq	FASTQ	DNA	1018526	109113824	35	107.1	151
P2-6080-Q-virome_R2.fastq	FASTQ	DNA	1018526	109229682	35	107.2	151
P2-6080-Ids-virome_R1.fastq	FASTQ	DNA	830553	96425469	35	116.1	151
P2-6080-Ids-virome_R2.fastq	FASTQ	DNA	830553	96502866	35	116.2	151
P3-5566-I-virome_R1.fastq	FASTQ	DNA	869113	99885773	35	114.9	151
P3-5566-I-virome_R2.fastq	FASTQ	DNA	869113	99970075	35	115	151
P3-5566-Q-virome_R1.fastq	FASTQ	DNA	895461	99874844	35	111.5	151
P3-5566-Q-virome_R2.fastq	FASTQ	DNA	895461	99977651	35	111.6	151
P3-5566-Ids-virome_R1.fastq	FASTQ	DNA	1018301	117549835	35	115.4	151
P3-5566-Ids-virome_R2.fastq	FASTQ	DNA	1018301	117660650	35	115.5	151
P4-1728-I-virome_R1.fastq	FASTQ	DNA	999725	113844547	35	113.9	151
P4-1728-I-virome_R2.fastq	FASTQ	DNA	999725	113966250	35	114	151
P4-1728-Q-virome_R1.fastq	FASTQ	DNA	969928	104680445	35	107.9	151
P4-1728-Q-virome_R2.fastq	FASTQ	DNA	969928	104790573	35	108	151
P4-1728-Ids-virome_R1.fastq	FASTQ	DNA	983342	114042200	35	116	151
P4-1728-Ids-virome_R2.fastq	FASTQ	DNA	983342	114146622	35	116.1	151
P5-8986-I-virome_R1.fastq	FASTQ	DNA	758798	88563713	35	116.7	151
P5-8986-I-virome_R2.fastq	FASTQ	DNA	758798	88653215	35	116.8	151
P5-8986-Q-virome_R1.fastq	FASTQ	DNA	891602	106335392	35	119.3	151
P5-8986-Q-virome_R2.fastq	FASTQ	DNA	891602	106413799	35	119.4	151
P5-8986-Ids-virome_R1.fastq	FASTQ	DNA	903287	104366876	35	115.5	151
P5-8986-Ids-virome_R2.fastq	FASTQ	DNA	903287	104467635	35	115.7	151
P6-8992-I-virome_R1.fastq	FASTQ	DNA	873992	99076446	35	113.4	151
P6-8992-I-virome_R2.fastq	FASTQ	DNA	873992	99149221	35	113.4	151

P6-8992-Q-virome_R1.fastq	FASTQ	DNA	746119	86020732	35	115.3	151
P6-8992-Q-virome_R2.fastq	FASTQ	DNA	746119	86107475	35	115.4	151
P6-8992-Ids-virome_R1.fastq	FASTQ	DNA	1103322	124352570	35	112.7	151
P6-8992-Ids-virome_R2.fastq	FASTQ	DNA	1103322	124414225	35	112.8	151

Supplementary Table 2

Sample name	Raw PE reads	PE reads after QC filtering	Human PE reads removed	Total PE Reads Before Mapping/Assembly	Reads mapped to vOTUs	Percent reads mapped to vOTUs	Number of viral contigs (geNOMAD)	Viral Contigs > 1000 bp	Viral Contigs > 2000 bp	Viral Contigs > 5000 bp	Viral Contigs > 10000 bp	Viral Contigs > 20000 bp	Assembly Length Total	Mean Contig Length	Median Contig Length	Max Contig Length	CheckV Quality Not Determined	CheckV Low Quality	CheckV Medium Quality	CheckV High Quality	CheckV Complete	Sum >= Medium Quality
**Viral enrichment: U=Protocol A; Z=Protocol A.0.1 (ZymoBIOMICS DNA/RNA Mini kit); Q=Protocol A.0.2 (AllPrep Power Viral DNA/RNA kit)**																						
9092-U-virome_S11_L001	1664460	1417212	995	1416217	930944	65,73%	8	4	2	0	0	0	8455	1323,75	898	3530	0	3	1	0	0	1
9092-1Q-virome_S3_L001	1742277	1419592	3481	1416111	133310	9,41%	75	19	7	1	0	0	42758	980,56	584	6658	5	14	0	0	0	0
9092-2Z-virome_S1_L001	1456005	1181383	175	1181208	444112	37,60%	52	4	1	0	0	0	7369	668,0576923	542	3489	1	3	0	0	0	0
2820-U-virome_S14_L001	1691399	1447463	2336	1445127	1371965	94,94%	20	11	8	5	0	0	42773	2363,65	1269,5	6542	0	3	3	2	3	8
2820-2Q-virome_S8_L001	1460188	1261848	96	1261752	636006	50,41%	9	3	1	0	0	0	6527	1115,888889	712	4366	3	0	0	0	0	0
2820-2Z-virome_S2_L001	1697036	1510265	76	1510189	918230	60,80%	0	0	0	0	0	0	0	0	0	0	0	0	0	0	0	0
9324-U-virome_S12_L001	1600505	1213434	17666	1195768	311530	26,05%	264	39	15	3	1	0	93469	784,4583333	489,5	10620	1	29	6	3	0	9
9324-2Q-virome_S5_L001	1542093	1396271	91	1396180	643011	46,06%	42	6	1	0	0	0	8466	633,9285714	469	2533	5	1	0	0	0	0
9324-2Z-virome_S4_L001	1375155	1191969	1565	1190404	332656	27,94%	59	16	3	1	0	0	27371	876,7627119	616	5561	2	14	0	0	0	0
0168-U-virome_S13_L001	1538372	1329715	3111	1326604	1227563	92,53%	14	6	3	2	0	0	18928	1726,214286	886,5	6192	0	3	1	1	1	3
0168-1Q-virome_S7_L001	1499517	1297634	1343	1296291	645200	49,77%	66	8	0	0	0	0	9828	630,3030303	525,5	1467	3	5	0	0	0	0
0168-1Z-virome_S6_L001	1862747	1591820	2930	1588890	831976	52,36%	11	0	0	0	0	0	0	489,9090909	456	748	0	0	0	0	0	0
8466-U-virome_S17_L001	1555191	1356565	322	1356243	1321599	97,45%	27	15	10	4	0	0	43128	1874,703704	1148	5980	0	11	0	3	1	4
8466-1Q-virome_S10_L001	1673012	1367067	255	1366812	879194	64,32%	8	4	3	0	0	0	8937	1419	970,5	2672	0	4	0	0	0	0
8466-2Z-virome_S9_L001	1410253	1237346	767	1236579	753906	60,97%	0	0	0	0	0	0	0	0	0	0	0	0	0	0	0	0
**Viral enrichment strategy: AC=Protocol A (ultracentrifugation); BC=Protocol A.1 (PEG-6000)**																						
SAC1-viromeR_S55_L001	365571	331725	12	331713	325189	98,03%	5	4	2	2	0	0	13680	2841,8	1422	5564	0	2	0	2	0	2
SAC2-viromeR_S56_L001	368602	337085	11	337074	328975	97,60%	6	4	2	1	0	0	13183	2364,166667	1478,5	5367	0	2	1	1	0	2
SBC1-virome	307751	283223	112	283111	266540	94,15%	17	5	3	3	0	0	19494	1399,235294	418	5838	1	2	0	2	0	2
SBC2-virome	321036	293170	18	293152	279677	95,40%	5	5	3	3	0	0	18983	3796,6	5030	5754	1	2	0	2	0	2
**Amplification strategy: AC=Protocol A (PCR); BD=Protocol A.2 (MDA)**																						
SAC1-viromeR_S55_L001	365571	331725	12	331713	325189	98,03%	5	4	2	2	0	0	13680	2841,8	1422	5564	0	2	0	2	0	2
SAC2-viromeR_S56_L001	368602	337085	11	337074	328975	97,60%	6	4	2	1	0	0	13183	2364,166667	1478,5	5367	0	2	1	1	0	2
SAD1-viromeR_S61_L001	227262	213851	4	213847	180846	84,57%	20	16	14	11	0	0	78864	4062,9	5286,5	6733	1	2	1	1	11	13
SAD2-virome	260881	244483	11	244472	213371	87,28%	18	16	14	12	0	0	83223	4681,777778	5423,5	6733	1	2	1	0	12	13
**Library kit: DP=Protocol A (DNA Prep library kit); TSeq=Protocol A.3 (TruSeq Nano DNA library Prep)**																						
S1-5393-VIROME_DP_S1_L001	5131783	4617437	3105	4614332	4360696	94,50%	46	22	15	8	0	0	75776	1954,217391	887,5	6655	3	7	2	6	4	12
S1-5393-viromeTSeq*	1654933	1624596	1002	1623594	1130562	69,63%	117	38	26	7	0	0	119466	1382,350427	612	8694	3	22	5	5	3	13
S3-9504-VIROME_DP_S3_L001	4781559	4434669	2810	4431859	4341508	97,96%	26	16	13	3	1	0	63267	2655,269231	1789,5	14806	0	12	3	0	1	4
S3-9504-viromeTSeq*	1217574	1021570	685	1020885	710624	69,61%	61	22	12	5	1	0	69616	1496,311475	677	10134	0	17	4	0	1	5
S4-1876-VIROME_DP_S4_L001	4913300	4540936	316	4540620	4332574	95,42%	21	13	9	3	0	0	41326	2232,428571	1196	5962	0	5	6	1	1	8
S4-1876-viromeTSeq*	1694898	1639087	112	1638975	1407161	85,86%	41	15	8	4	0	0	48570	1559,170732	718	8267	0	9	4	1	1	6
14-2820AC-VIROME_DP_S5_LO	3608775	3218914	3015	3215899	3143958	97,76%	35	13	9	5	0	0	46951	1681,114286	663	6541	0	6	2	1	4	7
14-2820AC-viromeTSeq*	1390669	1333453	335	1333118	824375	61,84%	100	22	11	4	0	0	59140	1002,25	525,5	6210	0	14	4	3	1	8
15-8466AC-VIROME_DP_S6_LO	3811556	3541076	423	3540653	3434672	97,01%	30	22	20	13	3	1	157204	5397,933333	4744,5	40578	1	6	4	6	5	15
15-8466AC-viromeTSeq*	1350120	1321047	113	1320934	1127492	85,36%	55	35	20	9	0	0	110440	2241,727273	1372	8076	2	22	3	5	3	11
**DNA/RNA extraction kit: Q=Protocol A (QIAamp Viral RNA mini kit); I=Protocol A.4 (Purelink kit RNA/DNA mini kit); Ids=Protocol A.4.1 (Purelink kit RNA/DNA mini kit and cDNA second strand synthesis)**																						
P1-2635-I-virome	897795	739396	39988	699408	406678	58,15%	115	38	24	6	5	2	198787	2112,782609	676	39339	3	29	5	1	0	6
P1-2635-Q-virome	1020060	856378	3949	852429	43415	5,09%	80	14	2	0	0	0	21574	767,3375	628	2928	4	9	1	0	0	1
P1-2635-Ids-virome	651458	489337	48254	441083	240429	54,51%	94	39	22	8	4	2	207172	2551,117021	765,5	42339	4	29	2	4	0	6
P2-6080-I-virome	919576	834110	2049	832061	821915	98,78%	56	25	15	8	0	0	78780	1724,428571	759,5	6171	2	11	4	2	6	12
P2-6080-Q-virome	1018526	904308	414	903894	825108	91,28%	29	13	8	3	0	0	40410	1747,448276	877	6172	0	6	3	1	3	7
P2-6080-Ids-virome	830553	744404	4126	740278	720882	97,38%	65	41	33	12	0	0	151707	2576,615385	2092	7374	4	20	7	1	9	17
P3-5566-I-virome	869113	791244	924	790320	782322	98,99%	17	11	11	5	0	0	50585	3191,882353	3523	6569	0	1	5	1	4	10
P3-5566-Q-virome	895461	804130	770	803360	771268	96,01%	19	12	10	5	0	0	49695	2873,947368	2833	6224	0	3	5	1	3	9
P3-5566-Ids-virome	1018301	916744	2171	914573	905757	99,04%	20	13	11	6	0	0	56442	3013,25	3255,5	6507	0	2	5	1	5	11
P4-1728-I-virome	999725	908923	786	908137	902666	99,40%	9	6	5	3	0	0	24296	2873,666667	3293	5938	0	1	2	1	2	5
P4-1728-Q-virome	969928	880027	950	879077	845089	96,13%	7	5	5	3	0	0	21682	3219,857143	3354	5758	0	1	1	1	2	4
P4-1728-Ids-virome	983342	906062	1385	904677	898980	99,37%	7	6	5	3	0	0	24181	3566,714286	3502	5860	0	2	1	1	2	4
P5-8986-I-virome	758798	693210	3668	689542	677737	98,29%	16	12	6	5	0	0	42080	2784,5	1493	6714	2	5	0	1	4	5
P5-8986-Q-virome	891602	821949	2757	819192	754922	92,15%	18	8	6	3	0	0	30978	2069,944444	925	6289	1	2	2	0	3	5
P5-8986-Ids-virome	903287	815061	11596	803465	782067	97,34%	19	11	8	5	0	0	48241	2751	1755	6736	1	3	2	0	5	7
P6-8992-I-virome	873992	789156	787	788369	772482	97,98%	8	4	2	2	0	0	14732	2105,375	982	6098	0	2	0	0	2	2
P6-8992-Q-virome	746119	681266	599	680667	646363	94,96%	5	2	2	2	0	0	11931	2723,2	880	6053	0	0	0	1	1	2
P6-8992-Ids-virome	1103322	987145	1921	985224	953333	96,76%	10	4	3	2	0	0	16032	2050,2	955,5	6120	0	2	0	0	2	2
*TruSeq samples are SE reads																						

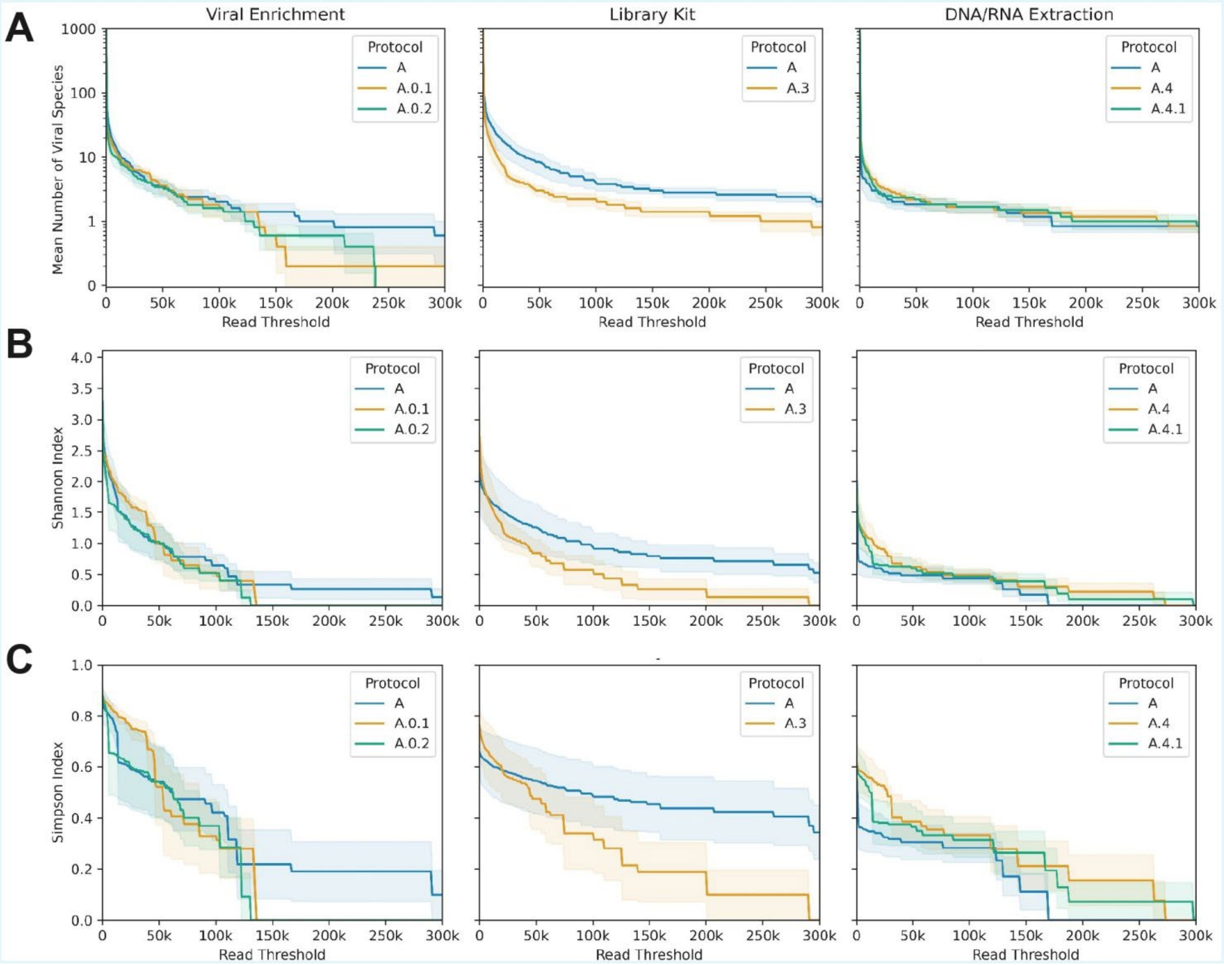

Figure S1: Diversity using read thresholds. Richness and diversity metrics of viral taxonomic assignments across protocols. Metrics were calculated using vOTUs, treated as species-level units, and calculated at increasing abundance read thresholds, disregarding any taxa below the required number of reads. Cutoffs from 0-300k with step sizes of 1000 were calculated. A) Species-level richness, defined as the number of vOTUs with read counts above each threshold. B) Species-level Shannon index. C) Species-level Simpson index. Protocols A.1 and A.2 were excluded from the analysis due to the small number of samples (n=2).

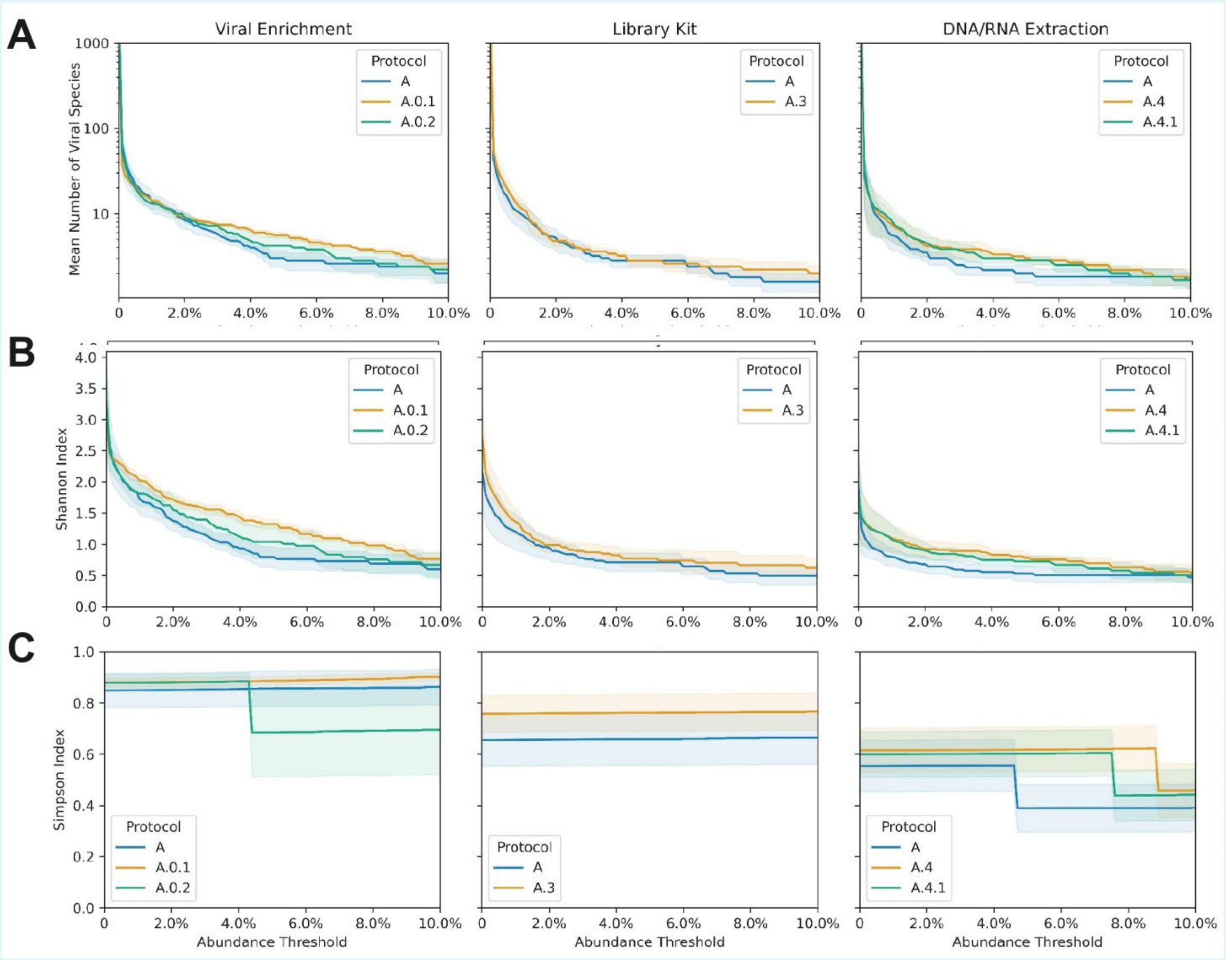

Figure S2: Low abundance diversity. Richness and diversity metrics of viral taxonomic assignments across protocols. Metrics were calculated using vOTUs, treated as species-level units, and calculated at increasing abundance thresholds, disregarding any taxa below the required abundance. Cutoffs from 0-10% with step sizes of 0.1% were calculated. A) Species-level richness, defined as the number of vOTUs with read counts above each threshold. B) Species-level Shannon index. C) Species-level Simpson index. Protocols A.1 and A.2 were excluded from the analysis due to the small number of samples (n=2).

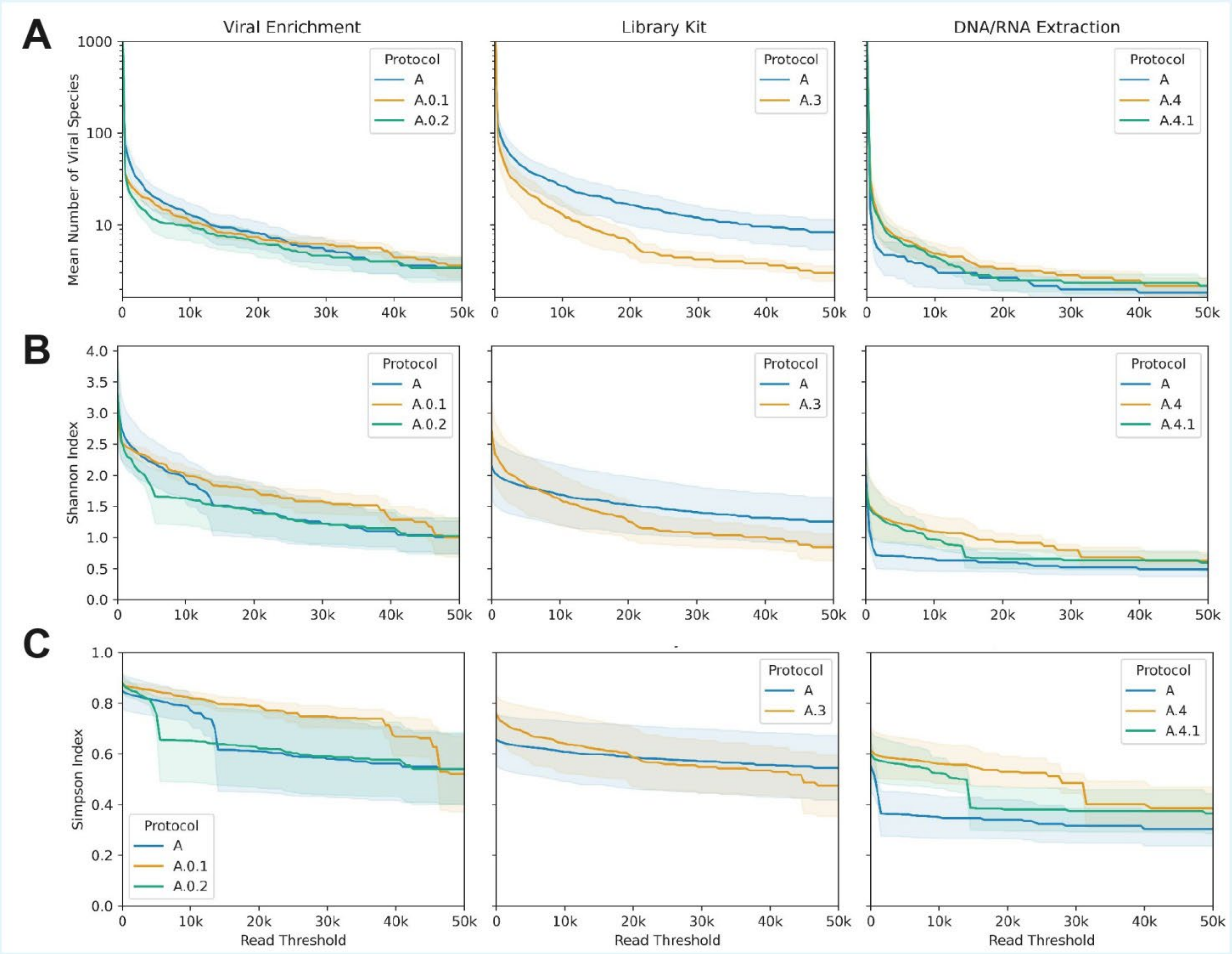

Figure S3: Low abundance diversity using read thresholds. Richness and diversity metrics of viral taxonomic assignments across protocols. Metrics were calculated using vOTUs, treated as species-level units, and calculated at increasing abundance read thresholds, disregarding any taxa below the required number of reads. Cutoffs from 0-50k with step sizes of 100 were calculated. A) Species-level richness, defined as the number of vOTUs with read counts above each threshold. B) Species-level Shannon index. C) Species-level Simpson index. Protocols A.1 and A.2 were excluded from the analysis due to the small number of samples (n=2).

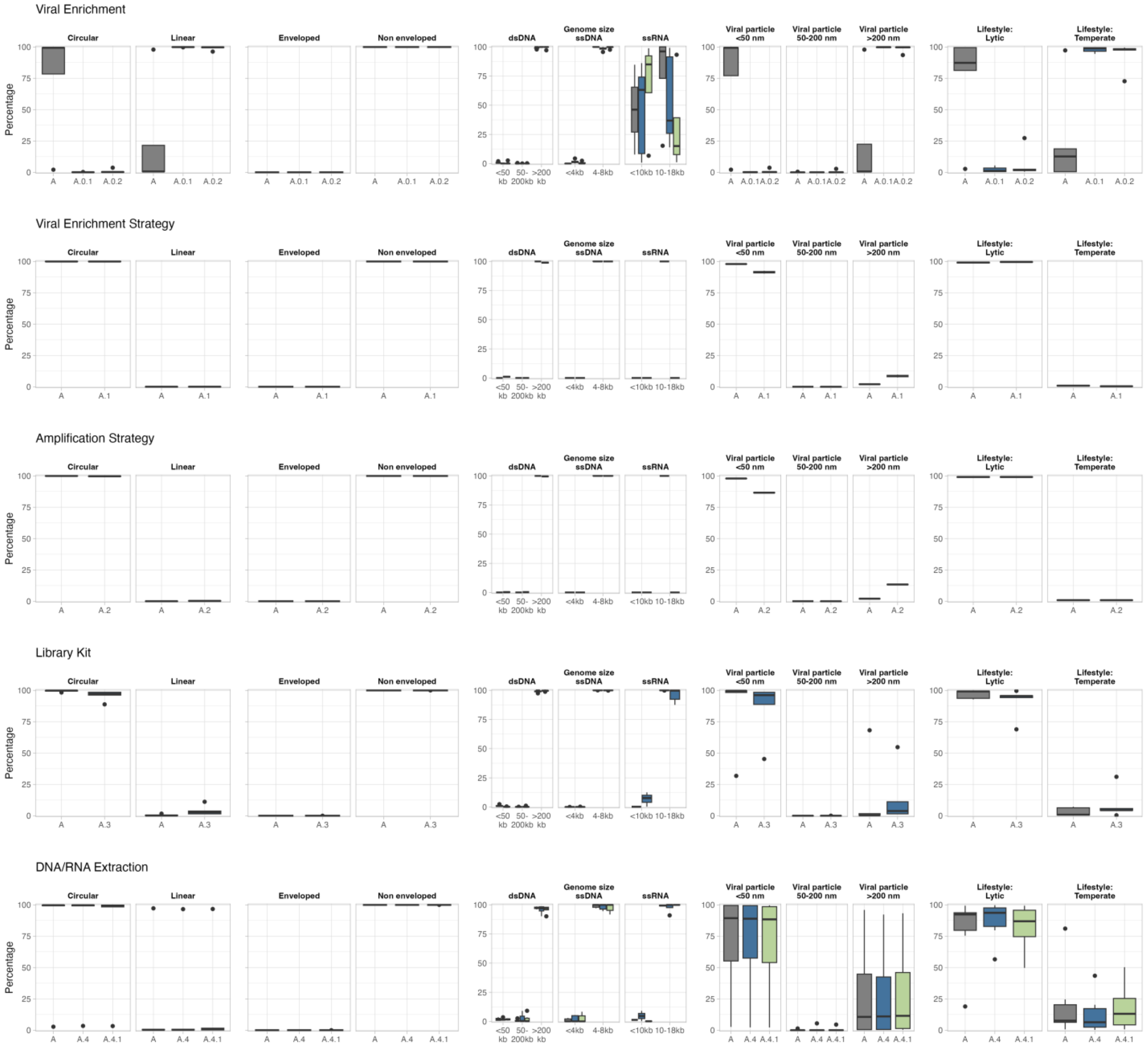

Figure S4: Structural and genomic characteristics of the viral families identified by each protocol. Boxplots for each protocol showing the characteristics of viruses: circular or linear genome, enveloped or non-enveloped structure, genome size, viral particle size (<50, 50-200 and >200 nm), and the proportion of lytic and temperate phages. The central line represents the median, box limits indicate Q1 and Q3, and whiskers extend to the lowest and highest non-outlier values (Q1-1.5 IQR and Q3+1.5 IQR).

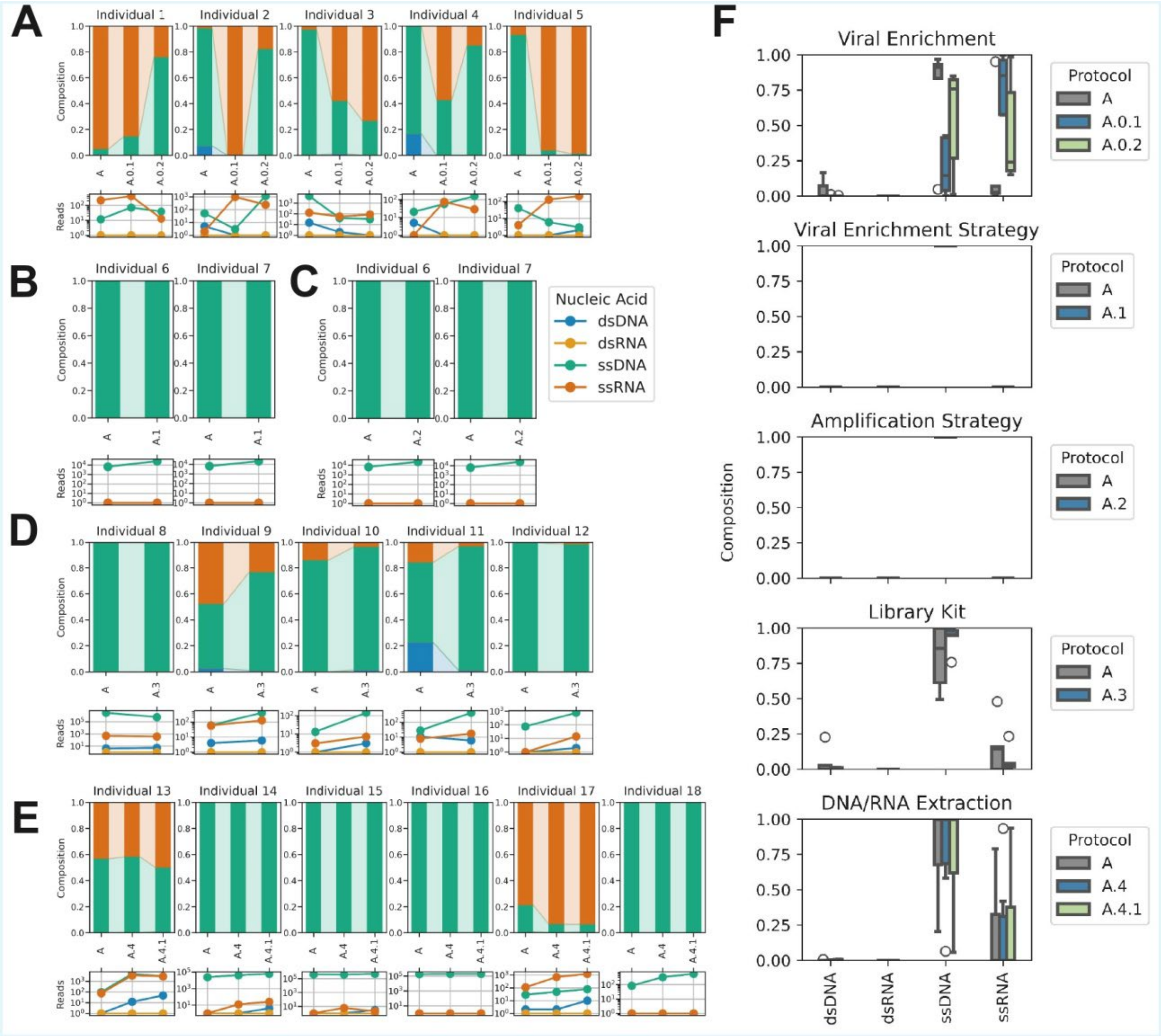

Figure S5: Composition of viruses by genome type for the protocols tested after excluding the *Microviridae* family and *Caudoviricetes* class. For A-E, the top figures represent the genome composition of individual samples, while the bottom one shows the reads of genome types (dsDNA, ssDNA and ssRNA). A) Viral enrichment, where protocol A corresponds to viral precipitation by ultracentrifugation and protocols A.0.1 and A.0.2 lack a viral enrichment step (nucleic acids were directly extracted using ZymoBIOMICS DNA/RNA Mini kit or AllPrep Power Viral DNA/RNA kit, respectively). B) Viral enrichment strategy, where protocol A corresponds to ultracentrifugation and protocol A.1 to PEG-600-based precipitation. C) Amplification strategy, where protocol A corresponds to PCR-SISPA and protocol A.2 to MDA. D) Library preparation kit, where protocol A corresponds to the DNA Prep (M) Tagmentation kit and protocol A.3 to the TruSeq Nano DNA Library Prep kit. E) DNA/RNA extraction, where protocol A corresponds to the QIAamp Viral RNA Mini kit, protocol A.4 to the PureLink Viral RNA/DNA Mini kit, and protocol A.4.1 to PureLink Viral RNA/DNA Mini kit followed by the cDNA second-strand synthesis. F) Boxplots showing the genome composition for the protocols. The central line represents the median, box limits indicate the Q1 and Q3 quartiles, and whiskers extend to Q1-1.5 IQR and Q3+1.5 IQR, marking the lowest and highest non-outlier values.

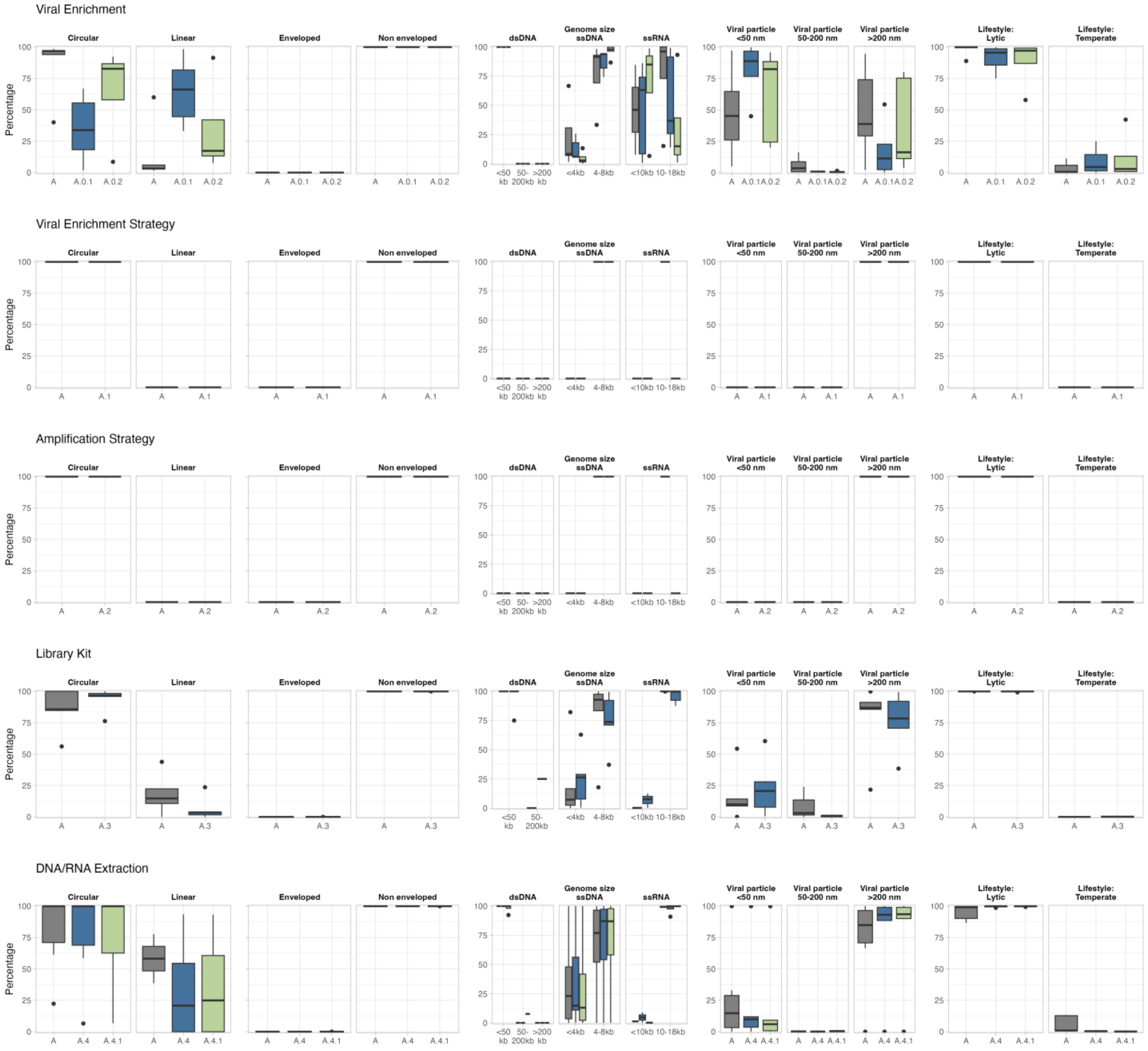

Figure S6: Structural and genomic characteristics of the viral families after excluding the *Microviridae* family and *Caudoviricetes* class, identified by each protocol. Boxplots for each protocol showing the characteristics of viruses: circular or linear genome, enveloped or non-enveloped structure, genome size, viral particle size (<50, 50-200 and >200 nm), and the proportion of lytic and temperate phages. The central line represents the median, box limits indicate Q1 and Q3, and whiskers extend to the lowest and highest non-outlier values (Q1-1.5 IQR and Q3+1.5 IQR).
