# Supplementary material for: A comparison of methods for the optimal recovery of the human fecal virome
Source: ISME Commun. 2026 Apr 11;6(1):ycag090. doi: 10.1093/ismeco/ycag090 (PMC13155102; doi:10.1093/ismeco/ycag090)
Supplement: Suppl_material_updated_ycag090_TableS2 [file suppl_material_updated_ycag090_tables2.pdf]

Supplementary Table 2

Sample name	Raw PE reads	PE reads after QC filtering	Human PE reads removed	Total PE Reads Before Mapping/Assembly	Reads mapped to vOTUs	Percent reads mapped to vOTUs	Number of viral contigs (geNOMAD)	Viral Contigs > 1000 bp	Viral Contigs > 2000 bp	Viral Contigs > 5000 bp	Viral Contigs > 10000 bp	Viral Contigs > 20000 bp	Assembly Length Total	Mean Contig Length	Median Contig Length	Max Contig Length	CheckV Quality Not Determined	CheckV Low Quality	CheckV Medium Quality	CheckV High Quality	CheckV Complete	Sum >= Medium Quality
**Viral enrichment: U=Protocol A; Z=Protocol A.0.1 (ZymoBIOMICS DNA/RNA Mini kit); Q=Protocol A.0.2 (AllPrep Power Viral DNA/RNA kit)**																						
9092-U-virome_S11_L001	1664460	1417212	995	1416217	930944	65, 73%	8	4	2	0	0	0	8455	1323, 75	898	3530	0	3	1	0	0	1
9092-1Q-virome_S3_L001	1742277	1419592	3481	1416111	133310	9, 41%	75	19	7	1	0	0	42758	980, 56	584	6658	5	14	0	0	0	0
9092-2Z-virome_S1_L001	1456005	1181383	175	1181208	444112	37, 60%	52	4	1	0	0	0	7369	668, 0576923	542	3489	1	3	0	0	0	0
2820-U-virome_S14_L001	1691399	1447463	2336	1445127	1371965	94, 94%	20	11	8	5	0	0	42773	2363, 65	1269, 5	6542	0	3	3	2	3	8
2820-2Q-virome_S8_L001	1460188	1261848	96	1261752	636006	50, 41%	9	3	1	0	0	0	6527	1115, 888889	712	4366	3	0	0	0	0	0
2820-2Z-virome_S2_L001	1697036	1510265	76	1510189	918230	60, 80%	0	0	0	0	0	0	0	0	0	0	0	0	0	0	0	0
9324-U-virome_S12_L001	1600505	1213434	17666	1195768	311530	26, 05%	264	39	15	3	1	0	93469	784, 4583333	489, 5	10620	1	29	6	3	0	9
9324-2Q-virome_S5_L001	1542093	1396271	91	1396180	643011	46, 06%	42	6	1	0	0	0	8466	633, 9285714	469	2533	5	1	0	0	0	0
9324-2Z-virome_S4_L001	1375155	1191969	1565	1190404	332656	27, 94%	59	16	3	1	0	0	27371	876, 7627119	616	5561	2	14	0	0	0	0
0168-U-virome_S13_L001	1538372	1329715	3111	1326604	1227563	92, 53%	14	6	3	2	0	0	18928	1726, 214286	886, 5	6192	0	3	1	1	1	3
0168-1Q-virome_S7_L001	1499517	1297634	1343	1296291	645200	49, 77%	66	8	0	0	0	0	9828	630, 3030303	525, 5	1467	3	5	0	0	0	0
0168-1Z-virome_S6_L001	1862747	1591820	2930	1588890	831976	52, 36%	11	0	0	0	0	0	0	489, 9090909	456	748	0	0	0	0	0	0
8466-U-virome_S17_L001	1555191	1356565	322	1356243	1321599	97, 45%	27	15	10	4	0	0	43128	1874, 703704	1148	5980	0	11	0	3	1	4
8466-1Q-virome_S10_L001	1673012	1367067	255	1366812	879194	64, 32%	8	4	3	0	0	0	8937	1419	970, 5	2672	0	4	0	0	0	0
8466-2Z-virome_S9_L001	1410253	1237346	767	1236579	753906	60, 97%	0	0	0	0	0	0	0	0	0	0	0	0	0	0	0	0
**Viral enrichment strategy: AC=Protocol A (ultracentrifugation); BC=Protocol A.1 (PEG-6000)**																						
SAC1-viromeR_S55_L001	365571	331725	12	331713	325189	98, 03%	5	4	2	2	0	0	13680	2841, 8	1422	5564	0	2	0	2	0	2
SAC2-viromeR_S56_L001	368602	337085	11	337074	328975	97, 60%	6	4	2	1	0	0	13183	2364, 166667	1478, 5	5367	0	2	1	1	0	2
SBC1-virome	307751	283223	112	283111	266540	94, 15%	17	5	3	3	0	0	19494	1399, 235294	418	5838	1	2	0	2	0	2
SBC2-virome	321036	293170	18	293152	279677	95, 40%	5	5	3	3	0	0	18983	3796, 6	5030	5754	1	2	0	2	0	2
**Amplification strategy: AC=Protocol A (PCR); BD=Protocol A.2 (MDA)**																						
SAC1-viromeR_S55_L001	365571	331725	12	331713	325189	98, 03%	5	4	2	2	0	0	13680	2841, 8	1422	5564	0	2	0	2	0	2
SAC2-viromeR_S56_L001	368602	337085	11	337074	328975	97, 60%	6	4	2	1	0	0	13183	2364, 166667	1478, 5	5367	0	2	1	1	0	2
SAD1-viromeR_S61_L001	227262	213851	4	213847	180846	84, 57%	20	16	14	11	0	0	78864	4062, 9	5286, 5	6733	1	2	1	1	11	13
SAD2-virome	260881	244483	11	244472	213371	87, 28%	18	16	14	12	0	0	83223	4681, 777778	5423, 5	6733	1	2	1	0	12	13
**Library kit: DP=Protocol A (DNA Prep library kit); TSeq=Protocol A.3 (TruSeq Nano DNA library Prep)**																						
S1-5393-VIROME_DP_S1_L001	5131783	4617437	3105	4614332	4360696	94, 50%	46	22	15	8	0	0	75776	1954, 217391	887, 5	6655	3	7	2	6	4	12
S1-5393-viromeTSeq*	1654933	1624596	1002	1623594	1130562	69, 63%	117	38	26	7	0	0	119466	1382, 350427	612	8694	3	22	5	5	3	13
S3-9504-VIROME_DP_S3_L001	4781559	4434669	2810	4431859	4341508	97, 96%	26	16	13	3	1	0	63267	2655, 269231	1789, 5	14806	0	12	3	0	1	4
S3-9504-viromeTSeq*	1217574	1021570	685	1020885	710624	69, 61%	61	22	12	5	1	0	69616	1496, 311475	677	10134	0	17	4	0	1	5
S4-1876-VIROME_DP_S4_L001	4913300	4540936	316	4540620	4332574	95, 42%	21	13	9	3	0	0	41326	2232, 428571	1196	5962	0	5	6	1	1	8
S4-1876-viromeTSeq*	1694898	1639087	112	1638975	1407161	85, 86%	41	15	8	4	0	0	48570	1559, 170732	718	8267	0	9	4	1	1	6
14-2820AC-VIROME_DP_S5_L0	3608775	3218914	3015	3215899	3143958	97, 76%	35	13	9	5	0	0	46951	1681, 114286	663	6541	0	6	2	1	4	7
14-2820AC-viromeTSeq*	1390669	1333453	335	1333118	824375	61, 84%	100	22	11	4	0	0	59140	1002, 25	525, 5	6210	0	14	4	3	1	8
15-8466AC-VIROME_DP_S6_L0	3811556	3541076	423	3540653	3434672	97, 01%	30	22	20	13	3	1	157204	5397, 933333	4744, 5	40578	1	6	4	6	5	15
15-8466AC-viromeTSeq*	1350120	1321047	113	1320934	1127492	85, 36%	55	35	20	9	0	0	110440	2241, 727273	1372	8076	2	22	3	5	3	11
**DNA/RNA extraction kit: Q=Protocol A (QIAamp Viral RNA mini kit); I=Protocol A.4 (Purelink kit RNA/DNA mini kit); Ids=Protocol A.4.1 (Purelink kit RNA/DNA mini kit and cDNA second strand synthesis)**																						
P1-2635-I-virome	897795	739396	39988	699408	406678	58, 15%	115	38	24	6	5	2	198787	2112, 782609	676	39339	3	29	5	1	0	6
P1-2635-Q-virome	1020060	856378	3949	852429	43415	5, 09%	80	14	2	0	0	0	21574	767, 3375	628	2928	4	9	1	0	0	1
P1-2635-Ids-virome	651458	489337	48254	441083	240429	54, 51%	94	39	22	8	4	2	207172	2551, 117021	765, 5	42339	4	29	2	4	0	6
P2-6080-I-virome	919576	834110	2049	832061	821915	98, 78%	56	25	15	8	0	0	78780	1724, 428571	759, 5	6171	2	11	4	2	6	12
P2-6080-Q-virome	1018526	904308	414	903894	825108	91, 28%	29	13	8	3	0	0	40410	1747, 448276	877	6172	0	6	3	1	3	7
P2-6080-Ids-virome	830553	744404	4126	740278	720882	97, 38%	65	41	33	12	0	0	151707	2576, 615385	2092	7374	4	20	7	1	9	17
P3-5566-I-virome	869113	791244	924	790320	782322	98, 99%	17	11	11	5	0	0	50585	3191, 882353	3523	6569	0	1	5	1	4	10
P3-5566-Q-virome	895461	804130	770	803360	771268	96, 01%	19	12	10	5	0	0	49695	2873, 947368	2833	6224	0	3	5	1	3	9
P3-5566-Ids-virome	1018301	916744	2171	914573	905757	99, 04%	20	13	11	6	0	0	56442	3013, 25	3255, 5	6507	0	2	5	1	5	11
P4-1728-I-virome	999725	908923	786	908137	902666	99, 40%	9	6	5	3	0	0	24296	2873, 666667	3293	5938	0	1	2	1	2	5
P4-1728-Q-virome	969928	880027	950	879077	845089	96, 13%	7	5	5	3	0	0	21682	3219, 857143	3354	5758	0	1	1	1	2	4
P4-1728-Ids-virome	983342	906062	1385	904677	898980	99, 37%	7	6	5	3	0	0	24181	3566, 714286	3502	5860	0	2	1	1	2	4
P5-8986-I-virome	758798	693210	3668	689542	677737	98, 29%	16	12	6	5	0	0	42080	2784, 5	1493	6714	2	5	0	1	4	5
P5-8986-Q-virome	891602	821949	2757	819192	754922	92, 15%	18	8	6	3	0	0	30978	2069, 944444	925	6289	1	2	2	0	3	5
P5-8986-Ids-virome	903287	815061	11596	803465	782067	97, 34%	19	11	8	5	0	0	48241	2751	1755	6736	1	3	2	0	5	7
P6-8992-I-virome	873992	789156	787	788369	772482	97, 98%	8	4	2	2	0	0	14732	2105, 375	982	6098	0	2	0	0	2	2
P6-8992-Q-virome	746119	681266	599	680667	646363	94, 96%	5	2	2	2	0	0	11931	2723, 2	880	6053	0	0	0	1	1	2
P6-8992-Ids-virome	1103322	987145	1921	985224	953333	96, 76%	10	4	3	2	0	0	16032	2050, 2	955, 5	6120	0	2	0	0	2	2
*TruSeq samples are SE reads																						
